# RGS19 activates the MYH9/β-catenin/c-Myc positive feedback loop in hepatocellular carcinoma

**DOI:** 10.1038/s12276-024-01244-9

**Published:** 2024-06-03

**Authors:** Shanjia Ke, Shounan Lu, Yanan Xu, Miaoyu Bai, Hongjun Yu, Bing Yin, Chaoqun Wang, Zhigang Feng, Zihao Li, Jingjing Huang, Xinglong Li, Baolin Qian, Yongliang Hua, Yao Fu, Bei Sun, Yaohua Wu, Yong Ma

**Affiliations:** 1 Department of Minimally Invasive Hepatic Surgery, The First Affiliated Hospital of Harbin Medical University, Harbin, China; 2 Key Laboratory of Hepatosplenic Surgery, Ministry of Education, The First Affiliated Hospital of Harbin Medical University, Harbin, China; 3 Department of Hepatopancreatobiliary Surgery, Affiliated Hangzhou First People’s Hospital, Zhejiang University School of Medicine, Hangzhou, China; 4 Department of Hepatobiliary Surgery, the Second Affiliated Hospital of Army Medical University, Chongqing, China; 5 The First Department of General Surgery, Affiliated Hospital of Inner Mongolia Minzu University, Tongliao, China; 6 Department of Thyroid Surgery, The First Affiliated Hospital of Harbin Medical University, Harbin, China; 7 Department of Pediatric Surgery, The First Affiliated Hospital of Harbin Medical University, Harbin, China; 8 Department of Ultrasound, The First Affiliated Hospital of Harbin Medical University, Harbin, China

**Keywords:** Oncogenes, Ubiquitylation

## Abstract

Hepatocellular carcinoma (HCC) is one of the most common fatal cancers worldwide, and the identification of novel treatment targets and prognostic biomarkers is urgently needed because of its unsatisfactory prognosis. Regulator of G-protein signaling 19 (RGS19) is a multifunctional protein that regulates the progression of various cancers. However, the specific function of RGS19 in HCC remains unclear. The expression of RGS19 was determined in clinical HCC samples. Functional and molecular biology experiments involving RGS19 were performed to explore the potential mechanisms of RGS19 in HCC. The results showed that the expression of RGS19 is upregulated in HCC tissues and is significantly associated with poor prognosis in HCC patients. RGS19 promotes the proliferation and metastasis of HCC cells in vitro and in vivo. Mechanistically, RGS19, via its RGS domain, stabilizes the MYH9 protein by directly inhibiting the interaction of MYH9 with STUB1, which has been identified as an E3 ligase of MYH9. Moreover, RGS19 activates β-catenin/c-Myc signaling via MYH9, and RGS19 is also a transcriptional target gene of c-Myc. A positive feedback loop formed by RGS19, MYH9, and the β-catenin/c-Myc axis was found in HCC. In conclusion, our research revealed that competition between RGS19 and STUB1 is a critical mechanism of MYH9 regulation and that the RGS19/MYH9/β-catenin/c-Myc feedback loop may represent a promising strategy for HCC therapy.

## RGS19 in HCC: Key Player in Poor Prognosis Mechanisms

Liver cancer is a worldwide health issue, with a type called hepatocellular carcinoma making up over 90% of cases. Researchers aimed to understand the biological workings of HCC to better predict patient outcomes. They studied the regulator of G protein signaling family, RGS19, a protein linked to several cancers’ progression. The study involved 100 HCC patients and used methods like gene expression analysis, cell culture experiments, and animal models. They found that RGS19 is overproduced in HCC tissues and high production is linked to poor survival rates. They also found RGS19 encourages growth and spread of HCC cells by interacting with another protein, MYH9, and activating cancer-related signaling pathway. The findings suggest that RGS19 could be a potential diagnostic marker and treatment target for HCC. The study also emphasizes the need for more research on RGS proteins’ role in cancer.

This summary was initially drafted using artificial intelligence, then revised and fact-checked by the author.

## Introduction

Primary liver cancer remains a major global health threat, and its incidence is increasing worldwide. It is estimated that the number of patients with liver cancer will reach more than 1 million by 2025^1,2^. Hepatocellular carcinoma (HCC) accounts for more than 90% of primary liver cancers^3^. The major risk factors for HCC include hepatitis virus infection, metabolic disorders associated with liver disease and excessive alcohol consumption^4^. Hence, elucidating the potential biological mechanisms of HCC is crucial for improving the prognosis of patients with HCC.

The regulator of G protein signaling (RGS) family participates in terminating G protein signaling by enhancing GTPase activity^5^. Abnormal expression of RGS proteins has been reported to be associated with various human diseases^6–8^. RGS19 (also known as G_α_-interacting protein, GAIP) acts as a GTPase activating protein (GAP) for G_αi1-3_ and G_α0_^9^. In addition, RGS19 performs multiple functions via its protein domain. For example, the C-terminus of RGS19 can interact with GIPC (GAIP-interacting protein C-terminus), and this interaction links RGS19 to various signaling pathways^10^. A previous study revealed that RGS19 participates in the regulation of cellular proliferation, and this function is apparently independent of its GAP activity^11^. In addition, the expression of RGS19 has been significantly associated with carcinogenesis in various cancers. For example, elevated RGS19 expression is correlated with poor prognosis in prostate cancer and ovarian cancer patients^12,13^. RGS19 also promotes the proliferation of bladder cancer cells in vitro and is significantly associated with poor patient prognosis^14^. However, the precise role of RGS19 and its potential molecular mechanisms in HCC are still unknown.

MYH9, also known as non-muscle myosin heavy chain IIA, has been reported to participate in multiple biological processes, including cell proliferation, invasion and polarity^15,16^. Although the expression of MYH9 is positively associated with a favorable prognosis in melanoma patients^17^, MYH9 has been reported to play a carcinogenic role in HCC, gastric cancer, colorectal cancer and pancreatic cancer^15,16,18,19^. Apart from transcriptional activation, how MYH9 is regulated in HCC remains to be clearly elucidated.

The Wnt/β-catenin pathway is a highly complex and conserved signaling pathway that regulates hepatobiliary development and liver homeostasis. Therefore, abnormal activation of the Wnt/β-catenin pathway is significantly associated with liver malignancies, such as HCC and cholangiocarcinoma, and other liver diseases, such as liver fibrosis and steatosis^20,21^. β-Catenin is a crucial component of the Wnt signaling cascade. When β-catenin accumulates in the cytoplasm and further translocates into the nucleus, it can regulate the expression of target genes by recruiting coactivators and transcription factors^22,23^. C-Myc, a downstream gene of β-catenin, contributes to the hallmarks of cancer, such as proliferation, self-renewal, cell survival, genome instability and immune evasion^24–26^. c-Myc amplification is found in 18–25% of human HCC samples^27^. In conclusion, targeting the β-catenin/c-Myc axis might be a promising treatment strategy for HCC patients.

To the best of our knowledge, this is the first study to show that RGS19 is an oncogene that promotes the proliferation and metastasis of HCC cells by activating the β-catenin/c-Myc axis with the assistance of MYH9. Moreover, RGS19 is directly regulated by the c-Myc protein at the transcriptional level. Therefore, our study revealed an RGS19/MYH9/β-catenin/c-Myc regulatory circuit in HCC, supporting RGS19 as a novel diagnostic marker and treatment target in patients with HCC.

## Materials and methods

### Clinical samples and HCC cell lines

Tissues from patients who underwent HCC resection surgery at the First Affiliated Hospital of Harbin Medical University between August 2011 and September 2015 were collected. All 100 HCC patients who participated in this study provided written informed consent. This study was approved by the Research Ethics Committee of the First Affiliated Hospital of Harbin Medical University. The IRB number is 201823. The Chinese Academy of Science (Shanghai, China) provided the following human HCC cell lines: Huh7, HepG2, PLC/PRF/5, HCCLM3, Hep3B, and HEK-293T. The normal liver cell line WRL-68 was obtained from AcceGen (Fairfield, USA). DMEM or MEM supplemented with 10% fetal bovine serum was used as the cell culture medium. All cell lines were cultured at 37 °C in an incubator with 5% CO_2_. All cell lines were authenticated by the suppliers.

### Statistical analysis

Each experiment was performed three times. Statistical analysis was performed with SPSS 22.0 software and GraphPad Prism 8.0 software. The results are presented as the mean ± standard deviation (SD). Student’s t test or one-way or two-way ANOVA was used to compare the differences between independent samples. Correlations were analyzed by Pearson’s correlation and linear regression analysis. *P* < 0.05 was considered to indicate statistical significance.

### Additional methods

Details of all procedures are presented in the Supporting Information.

## Results

### Upregulated expression of RGS19 is associated with poor prognosis in patients with HCC

**Fig. 1:**
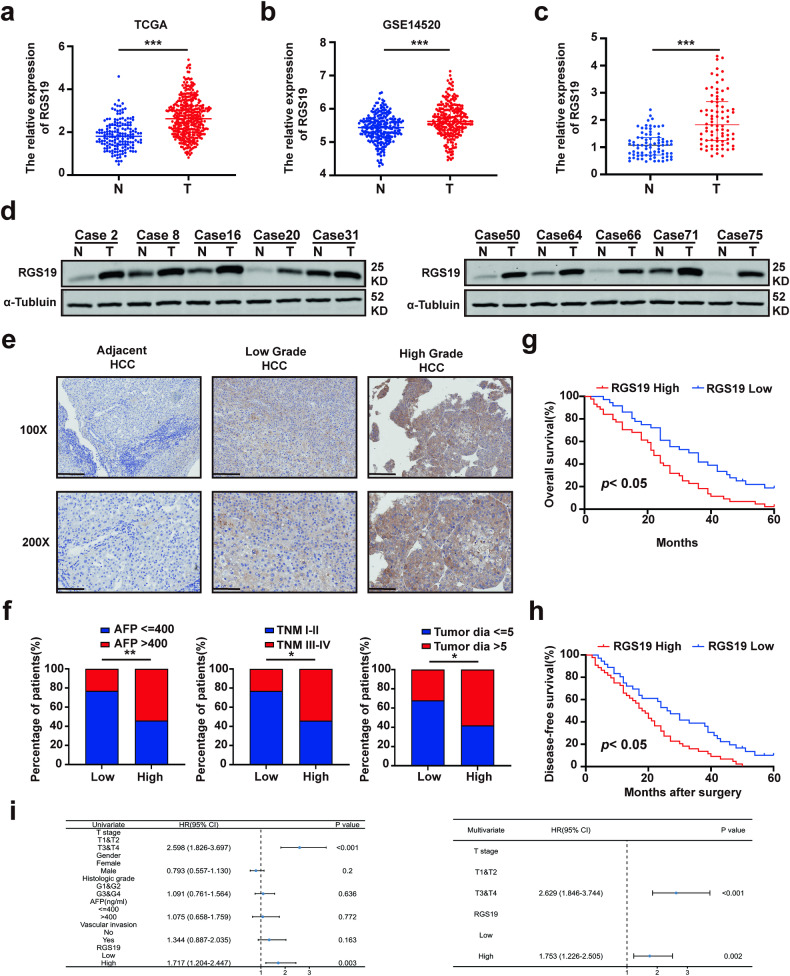
Increased expression of RGS19 is correlated with poor prognosis in patients with HCC. **a** RGS19 expression was determined in HCC tissues and corresponding noncancerous liver tissues using a TCGA dataset. **b** RGS19 expression was determined in HCC tissues and corresponding noncancerous liver tissues in the GSE14520 dataset. **c** The RGS19 mRNA level in HCC tissues and adjacent nontumor tissues was measured by real-time PCR (*n* = 80). **d** Representative images of RGS19 protein levels in 80 paired human HCC and adjacent tissues. **e** Typical images of RGS19 IHC staining in HCC and adjacent liver tissues. Scale bars: 100× = 200 μm; 200× = 100 μm. **f** Percentage of patients with high and low RGS19 expression among 97 HCC patients with different tumor sizes, TNM stages and AFP levels. **g**, **h** Kaplan‒Meier survival curves of OS and DFS for 97 HCC patients with relatively low and high RGS19 expression. **i** Univariate and multivariate Cox proportional hazards analyses of HCC patients. All experiments were performed three times, and the data are presented as the mean ± SD. *p < 0.05; **p < 0.01; ***p < 0.001. N: normal; T: tumor, TCGA: The Cancer Genome Atlas, TNM: tumor node metastasis, AFP: alpha fetoprotein, DFS: disease-free survival, OS: overall survival, IHC: immunohistochemistry.

First, we analyzed the expression of RGS19 in the TCGA (The Cancer Genome Atlas) and GEO (Gene Expression Omnibus) databases^28^. We found that the expression of RGS19 was elevated in HCC tissues compared with nontumor tissues (Fig. 1a, b). The upregulated expression of RGS19 in clinical HCC tissues compared with adjacent normal tissues was also validated by qPCR and Western blotting (Fig. 1c, d). In addition, 97 pairs of HCC tissues and adjacent noncancerous tissues were used to evaluate the differential expression of RGS19 and the associations between RGS19 expression and clinical parameters by IHC staining (Fig. 1e). The results indicated that increased RGS19 expression was positively associated with increased tumor size (*p* = 0.014), increased AFP levels (*p* = 0.003) and advanced TNM stage (*p* = 0.014) (Fig. 1f and Supplementary Table 1). K‒M survival analysis further suggested that compared with low RGS19 expression, high RGS19 expression was strongly associated with shorter overall survival (OS) and disease-free survival (DFS) in HCC patients (Fig. 1g, h). Similar results were obtained from the TCGA database (Supplementary Fig. 1a, b). Combined with univariate and multivariate Cox models from the TCGA database, the expression of RGS19 could serve as an independent prognostic factor for patients with HCC (Fig. 1i). Thus, these data demonstrated that the expression of RGS19 is upregulated in HCC and is positively correlated to poor survival in patients with HCC.

### RGS19 promotes HCC proliferation in vitro and in vivo

**Fig. 2:**
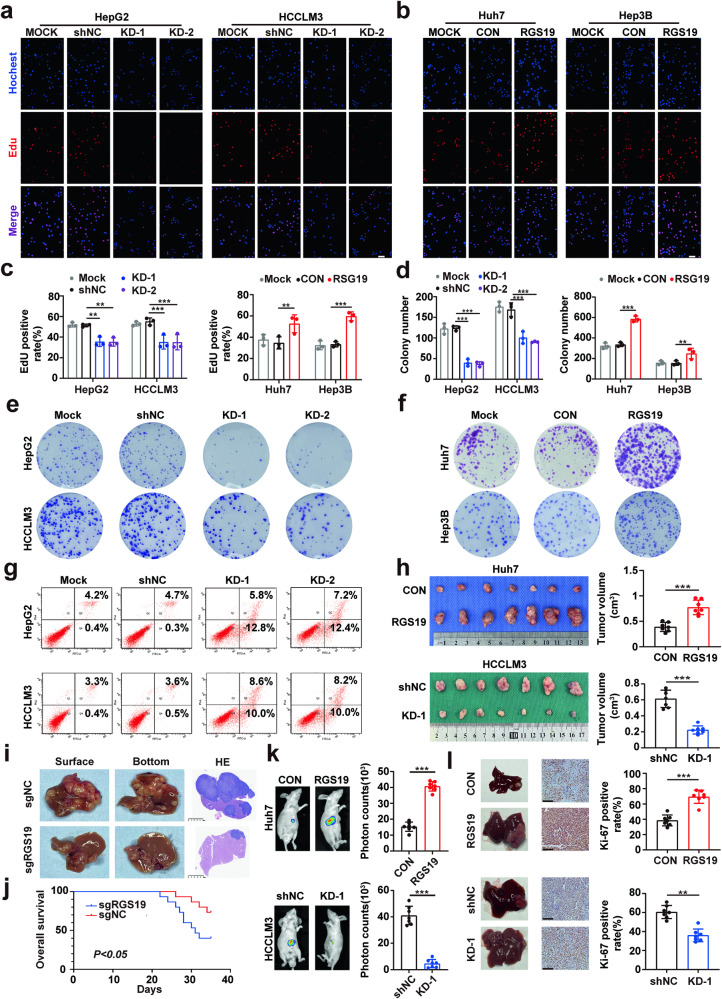
RGS19 promotes HCC cell proliferation and tumorigenesis in vitro and in vivo. **a**, **b** Typical images of the EdU assays in the indicated HCC cell lines. Scale bars: 100 μm. **c** Statistical analysis of the EdU assay results. **d** Statistical analysis of colony numbers. **e**, **f** Typical images of colony formation of the indicated HCC cell lines. **g** Effect of RGS19 knockdown on apoptosis in HepG2 and HCCLM3 cells. (h) Representative images of subcutaneous xenografts and tumor volume analysis of mice injected with the indicated HCC cells (*n* = 7/group). **i** Representative images of liver specimens and H&E staining of liver HCC nodules from C57BL/6 mice after hydrodynamic injection of sgNC/sgTP53/c-Myc or sgRGS19/sgTP53/c-Myc. Scale bars: 5 mm. **j** Kaplan‒Meier plot of the C57BL/6 mice OS data described above. **k** Bioluminescence images of mice with transfected Huh7 and HCCLM3 cells and analysis of photon counts. **l** Typical images of liver specimens with Ki-67 staining. Scale bars: 100 μm. All experiments were performed three times, and the data are presented as the mean ± SD. *p < 0.05; **p < 0.01; ***p < 0.001. EdU: 5-ethynyl-2’-deoxyuridine, sgNC: single-guide negative control, sgTP53: single-guide tumor protein53 (TP53), sgRGS19: single-guide RGS19.

Before the functional experiments, the mRNA and protein expression levels of RGS19 were evaluated in a normal liver cell line (WRL-68) and HCC cell lines (Supplementary Fig. 2a, b). RGS19-knockdown HepG2 and HCCLM3 cell lines and RGS19-overexpressing Huh7 and Hep3B cells were established because of their endogenous expression of RGS19. The efficiency of RGS19 lentiviral transfection was verified at the mRNA and protein levels (Supplementary Fig. 3a-d). Next, we found that RGS19 upregulation significantly increased the proliferative ability of Huh7 and Hep3B cells, whereas RGS19 silencing had the opposite effect on HepG2 and HCCLM3 cells according to 5-ethynyl-20-deoxyuridine (EdU) and CCK-8 assays (Fig. 2a–c and Supplementary Fig. 4a, b). Similarly, colony formation assays indicated that RGS19 overexpression in Huh7 and Hep3B cells significantly increased colony numbers, while RGS19 knockdown reduced colony numbers in HepG2 and HCCLM3 cells (Fig. 2d-f). In addition, the apoptosis rate of RGS19-silenced HCC cells was significantly greater than that of control cells (Fig. 2g and Supplementary Fig. 5). In addition to these assays, we constructed an RGS19^Mut^ plasmid in which the GAP functions of RGS19 were suppressed^29^. Surprisingly, according to the CCK-8 and colony formation assays, the overexpression of RGS19^Mut^ or RGS19^WT^ in Huh7 and Hep3B cells promoted the proliferation of HCC cells to a similar extent as that in the CON group (Supplementary Fig. 6a, b). These results indicated that RGS19 promotes the development of HCC cells independent of its GAP function. Next, to explore the biological functions of RGS19 in vivo, we established a subcutaneous xenograft mouse model. The results showed that the implantation of Huh7-RGS19 cells resulted in increased tumor volume, while the implantation of HCCLM3-shRGS19 cells impaired tumorigenesis in the nude mouse model (Fig. 2h). The number of TUNEL-positive cells in subcutaneous xenograft tumor sections was significantly greater in the KD-1 group than in the shNC group (Supplementary Fig. 7). To further confirm the pivotal role of RGS19 in tumor growth, we established a C57BL/6 HCC mouse model. After hydrodynamic injection, the vector/sgTP53/c-Myc/SB13 and sgRGS19/sgTP53/c-Myc/SB13 plasmids were used to construct the primary liver tumor model. The data showed that RGS19 knockout significantly decreased the formation of HCC and prolonged the survival of the mice (Fig. 2i, j). The number of TUNEL-positive cells in the tumor sections was significantly greater in the RGS19-knockout group than in the vector group (Supplementary Fig. 8). We also established a liver orthotopic nude mouse tumor model and evaluated the intensity of Ki-67 and TUNEL staining in liver tumor sections. The results were similar to those mentioned above. Bioluminescence imaging revealed that RGS19 overexpression promoted tumor growth, while RGS19 knockdown significantly inhibited tumor growth (Fig. 2k). Compared with those in the control group, the Huh7-RGS19 group showed larger orthotopic liver tumors, stronger Ki-67 staining and weaker TUNEL-positive staining, while the HCCLM3-shRGS19 group showed the opposite trend and more TUNEL-positive cells (Fig. 2l and Supplementary Fig. 9). Therefore, our results demonstrated that RGS19 promotes HCC growth both in vitro and in vivo.

### RGS19 enhances HCC metastasis in vitro and in vivo

Metastasis is a major threat for patients with HCC. Therefore, we next investigated the effect of RGS19 on the metastatic ability of HCC cells. Wound-healing experiments showed that upregulated RGS19 enhanced the migratory ability of Huh7 and Hep3B cells, but this effect was abrogated by knocking down RGS19 in HepG2 and HCCLM3 cells (Supplementary Fig. 10a–d). Transwell assays were performed to assess the invasion and migratory ability of HCC cells. The data showed that overexpression of RGS19 increased, whereas knockdown of RGS19 impaired, the invasion and migration ability of HCC cells (Supplementary Fig. 10e–h). A pulmonary metastasis mouse model was established for in vivo investigations. With bioluminescence imaging, we found that mice injected with Huh7-RGS19 cells, had more lung metastatic nodules than the respective control mice, and HCCLM3-shRGS19 cells exhibited the opposite phenomenon (Supplementary Fig. 10i, j). These results indicated that RGS19 is involved in promoting HCC metastasis.

### Direct interactions between RGS19 and MYH9

**Fig. 3:**
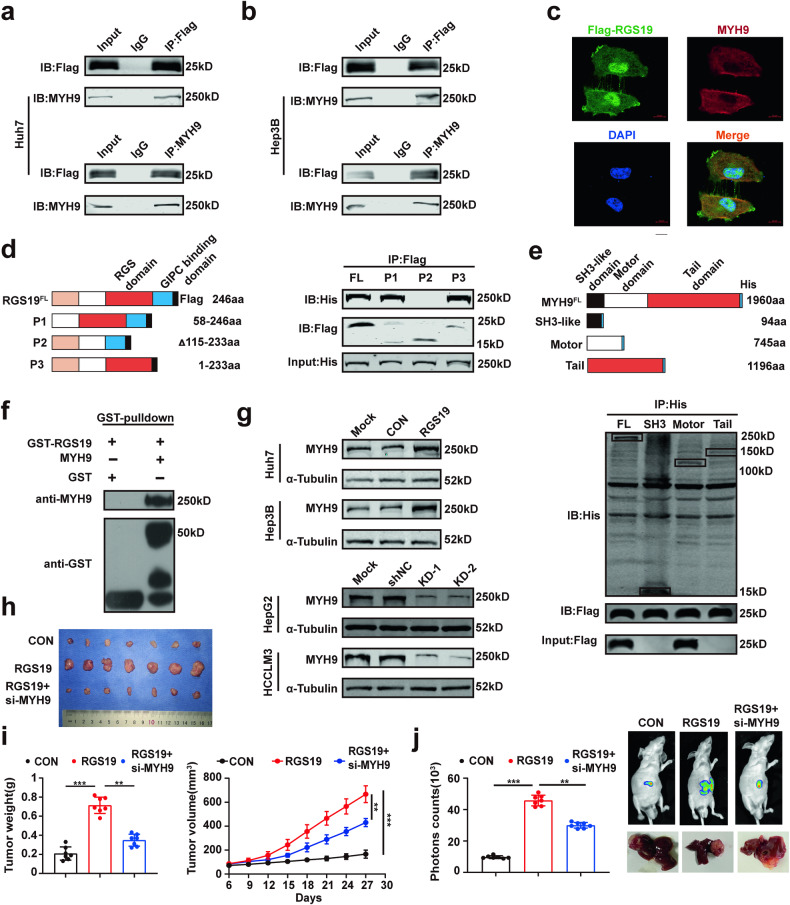
RGS19 directly interacts with MYH9. **a**, **b** Immunoprecipitation and WB analysis of the interaction between RGS19 and MYH9 in Huh7 and HHCLM3 cells transfected with FLAG-RGS19. **c** Confocal microscopy analysis showing the colocalization of RGS19 and MYH9 in the cytoplasm of Huh7 cells. Scale bars: 10 μm. **d** Full-length RGS19 and its truncated forms (P1, P2, and P3) with MYH9^His^ were co-transfected into HEK-293T cells. Lysates of 293 T cells were immunoprecipitated with an anti-Flag antibody (against RGS19). Immunoblot analysis was performed with an anti-His antibody (against MYH9). **e** Full-length MYH9 and its truncated forms (SH3-like, motor and tail domains) were co-transfected with RGS19^Flag^ into HEK-293T cells. Lysates of 293 T cells were immunoprecipitated with an anti-His antibody (against MYH9), and immunoblot analysis was performed with an anti-Flag antibody (against RSG19). **f** In vitro binding analysis of MYH9 and RGS19^GST^ via GST pulldown assays. **g** Western blot analysis of MYH9 expression in the indicated HCC cell lines. **h** Representative images of the subcutaneous xenografts in the indicated groups of mice (*n* = 7/group). **i** Analysis of subcutaneous xenograft weight and tumor growth. **j** The bioluminescence images of mice and typical images of orthotopic liver tumor model specimens (*n* = 7/group) and the analysis of photon counts of the model. All experiments were performed three times, and the data are presented as the mean ± SD. *p < 0.05; **p < 0.01; ***p < 0.001. GST: glutathione-s-transferase.

To explore how RGS19 regulates the functions of HCC, we performed co-IP and mass spectrometry (MS) assays to identify RGS19-binding proteins (Supplementary Table 4). The MS results showed that MYH9 is a potential candidate that can interact with RGS19. Co-IP assays were used to validate the interaction between RGS19 and MYH9 in HCC cells (Fig. 3a, b). The endogenous interaction between RGS19 and MYH9 was also validated in HepG2 and HCCLM3 cells (Supplementary Fig. 11). IF assays subsequently confirmed the colocalization of RGS19 and MYH9 in the cytoplasm of HCC cells (Fig. 3c). Additionally, molecular mapping experiments revealed that RGS19^P2^ was unable to interact with full-length MYH9 (Fig. 3d). In addition, we transfected the RGS19^P2^ plasmid into Huh7 cells and found that there was no interaction between RGS19^P2^ and MYH9 by IF assays (Supplementary Fig. 12). These results suggested that the RGS domain is necessary for the RGS19 protein to mediate binding with MYH9. Among the three major domains of the MYH9 protein, the motor domain of MYH9 was responsible for its association with RGS19 (Fig. 3e). Finally, we purified the GST-RGS19 protein in vitro, and glutathione S-transferase (GST) pull-down assays indicated that MYH9 was directly bound to GST-RGS19 (Fig. 3f). Taken together, these results suggested a direct interaction between RGS19 and MYH9.

### RGS19 regulates the proliferation of HCC cells by increasing MYH9 protein levels

After validating the interaction between RGS19 and MYH9, we next investigated the regulatory effect of RGS19 on MYH9 in HCC cells. WB analysis showed that RGS19 overexpression increased the protein levels of MYH9 in Huh7 and Hep3B cells, while the opposite trends were observed in HepG2-shRGS19 and HCCLM3-shRGS19 cells (Fig. 3g). Intriguingly, we found that the mRNA expression of MYH9 was not altered by regulating RGS19 expression in HCC cells (Supplementary Fig. 13a–d). These results suggested that RGS19 might regulate the expression of MYH9 at the posttranslational level. We further explored whether RGS19 promotes HCC tumorigenesis by regulating MYH9 in vitro and in vivo. The downregulation of MYH9 expression suppressed the proliferative ability of RGS19-overexpressing Huh7 cells, while the overexpression of MYH9 enhanced the proliferative ability of RGS19-knockdown cells, as determined by colony formation assays and CCK-8 assays (Supplementary Fig. 14a–d). Similar results were acquired in a mouse model with MYH9 knockdown in RGS19-overexpressing cells. Both bioluminescence imaging and tumor specimen analysis indicated that MYH9 knockdown suppressed the promotive effect of RGS19 overexpression on tumor growth (Fig. 3h–j). Taken together, these results demonstrated that MYH9 is crucial for RGS19-mediated HCC development.

### The interaction between RGS19 and MYH9 impairs proteasome-mediated MYH9 degradation

**Fig. 4:**
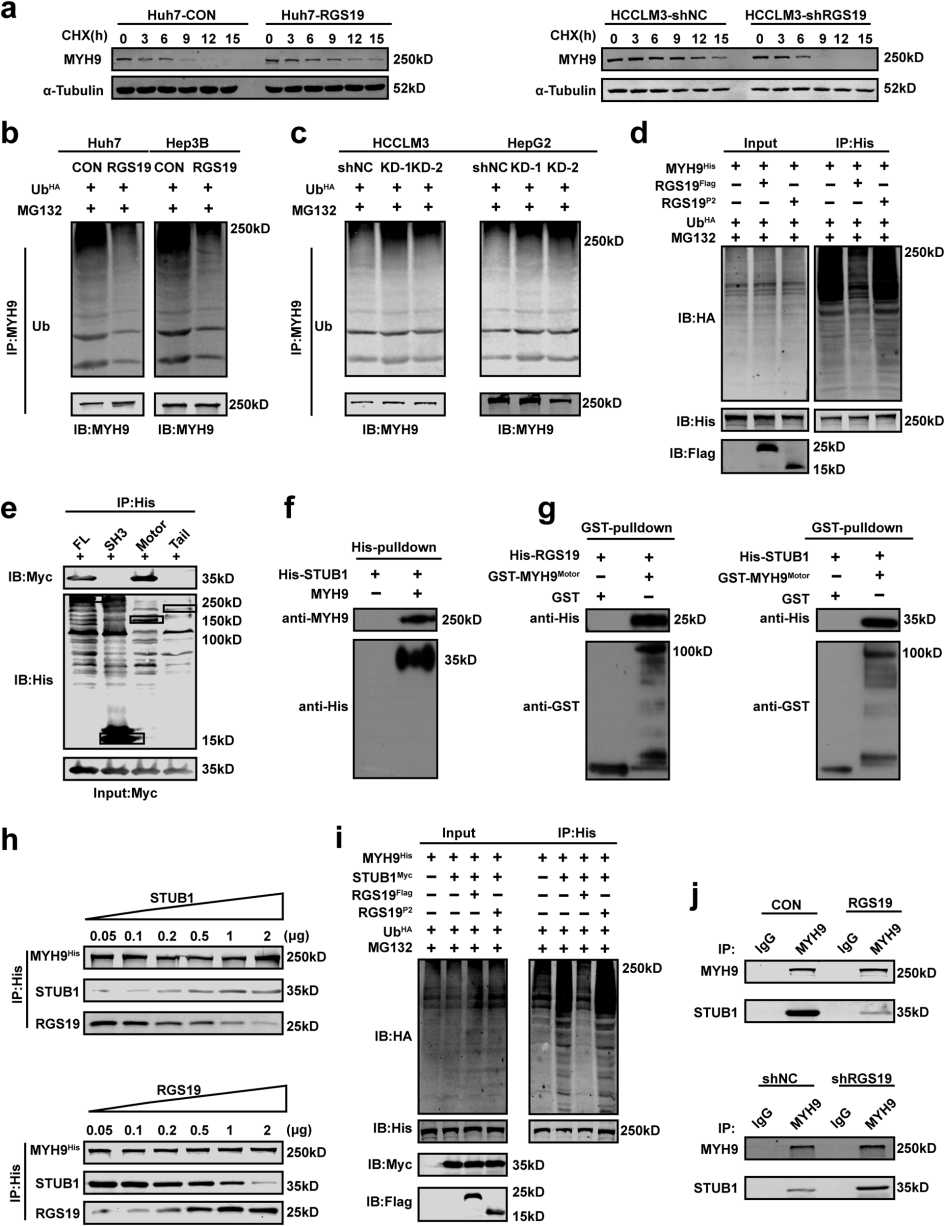
RGS19 stabilizes MYH9 by abrogating its STUB1-mediated degradation. **a** The effect of RGS19 overexpression on the half-life of MYH9 in Huh7 cells and of RGS19 silencing on the half-life of MYH9 in HCCLM3 cells treated with cycloheximide (CHX, 20 μmol). **b** Ubiquitination of MYH9 in Huh7 and Hep3B cells transfected with Con or Flag-RGS19 and treated with MG132 (100 nmol). **c** Ubiquitination of MYH9 after shNC or sh-RGS19 transfection and MG132 (100 nmol) treatment in HCCLM3 and HepG2 cells. **d** Ubiquitination of MYH9 with plasmids encoding RGS19^Flag^ or RGS19^P2^ (without the RGS domain), co-transfection of Ub^HA^ and MYH9^His^ and treatment with MG132 in HEK293T cells. **e** Full-length MYH9^His^ and its truncated form with STUB1^Myc^ were co-transfected into HEK-293T cells. Lysates of HEK293T cells were immunoprecipitated with an anti-His antibody, and immunoblot analysis was performed with an anti-Myc antibody (against STUB1). **f** In vitro binding analysis of MYH9 and STUB1^His^ via His pulldown assay. **g** Binding analysis of GST-MYH9^Motor^ and purified RGS19^His^ or STUB1^His^ using GST pulldown assays in vitro. **h** The indicated doses of purified RGS19 or STUB1 protein were added to the lysates of HEK-293T cells transfected with His-MYH9. Immunoprecipitation was performed with anti-His antibodies, and the results were obtained by WB analysis with the indicated antibodies. **i** Ubiquitination of MYH9 in HEK293T cells cotransfected with plasmids encoding RGS19^Flag^ or RGS19^P2^ (without the RGS domain) or treated with STUB1^Myc^, Ub^HA,^ MYH9^His^ or MG132. **j** Immunoprecipitation of MYH9 and STUB1 was detected in Huh7 cells transfected with vector or RGS19 plasmids, as shown in the upper panel, and immunoprecipitation of MYH9 and STUB1 was detected in HCCLM3 cells transfected with shNC or sh-RGS19 plasmids, as shown in the lower panel. All experiments were performed three times, and the data are presented as the mean ± SD. *p < 0.05; **p < 0.01; ***p < 0.001. shNC: short hairpin normal control; sh-RGS19: short hairpin-RGS19.

To investigate how RGS19 regulates the protein level of MYH9, we administered cycloheximide (CHX, 20 μM) to HCC cells, and the protein expression of MYH9 was monitored at different time points after CHX treatment. The results revealed that the half-life of the MYH9 protein was obviously longer in RGS19-overexpressing cells than in control cells, while the half-life of the MYH9 protein was shorter in HCCLM3-shRGS19 cells than in control cells (Fig. 4a). Because protein degradation mainly depends on the lysosome or proteasome pathway, we next investigated which pathway is responsible for MYH9 degradation. Chloroquine (CQ, a lysosome inhibitor) and MG132 (a proteasome inhibitor) were used in the subsequent experiments. Western blotting showed that MG132 strongly rescued the downregulation of MYH9 caused by RGS19 depletion, while CQ had no influence on MYH9 expression in HCC cells (Supplementary Fig. 15). Furthermore, elevated expression of RGS19 reduced the polyubiquitination of MYH9 in Huh7 and Hep3B cells, while the opposite trend was observed in HCCLM3-shRGS19 and HepG2-shRGS19 cells (Fig. 4b, c). In addition, we found that RGS19 overexpression markedly decreased the ubiquitination of MYH9, while the P2 truncation of RGS19 (without the RGS domain) had no influence on MYH9 ubiquitination in HEK293T cells (Fig. 4d). These results suggested that the interaction between RGS19 and MYH9 might be necessary for preventing the degradation of MYH9.

### RGS19 stabilizes the MYH9 protein by competitively abrogating the MYH9-STUB1 interaction

Considering that RGS19 has no deubiquitinating ability, we speculated that RGS19 modulates the level of MYH9 ubiquitination by preventing its interaction with the E3 ubiquitin ligase. A previous study demonstrated that STUB1, an E3 ubiquitin ligase, is involved in the degradation of MYH9 in colorectal cancer^15^. First, we identified the interaction between MYH9 and STUB1 and confirmed that STUB1 regulated the expression of MYH9 in HCC cells (Supplementary Fig. 16a, b). However, there were no interactions or correlations between RGS19 and STUB1, which suggested that RGS19, MYH9, and STUB1 did not form a triple-protein complex (Supplementary Fig. 16a–d). Subsequent molecular mapping experiments between MYH9 and STUB1 revealed that the motor domain of MYH9 was responsible for binding to STUB1 and that the U-box domain of STUB1 was necessary for binding to MYH9 (Fig. 4e and Supplementary Fig. 17). Next, a His pull-down assay validated the direct interaction between MYH9 and STUB1^His^ in vitro (Fig. 4f). We were surprised to find that RGS19 and STUB1 interact with the same domain of MYH9. To further investigate the direct interaction between RGS19 and STUB1 with the MYH9 motor domain in vitro, a GST pulldown assay was used to confirm that purified RGS19^His^ and the STUB1^His^ protein could directly bind to the GST-MYH9 motor domain (Fig. 4g). Therefore, we hypothesized that RGS19 influences the interaction between MYH9 and STUB1 and further prevents the STUB1-induced degradation of MYH9. In addition, when the amount of purified RGS19 protein was increased in a dose-dependent manner, the interaction between STUB1 and MYH9 gradually decreased, and the interaction even disappeared from the proteins pulled down by His-tagged MYH9. The associations between RGS19 and MYH9 were also attenuated by the addition of purified STUB1 protein in a dose-dependent manner (Fig. 4h). Moreover, in HEK293T cells, the overexpression of full-length RGS19 but not the P2 truncation of RGS19 suppressed the STUB1-mediated ubiquitination of MYH9 (Fig. 4i). Ultimately, we found that the overexpression of RGS19 significantly impaired the endogenous interaction between MYH9 and STUB1, while the knockdown of RGS19 enhanced the interaction between MYH9 and STUB1 in HCC cells (Fig. 4j). Similarly, we found that the interaction between MYH9 and RGS19 was enhanced when STUB1 was knocked down in Huh7 cells, while this interaction was impaired when STUB1 was overexpressed in HCCLM3 cells (Supplementary Fig. 18a, b). In summary, these data verified that RGS19 disrupts the MYH9-STUB1 complex by competitively interacting with the MYH9 motor domain and further maintaining the protein stability of MYH9.

### RGS19 activates the β-catenin/c-Myc axis via MYH9

**Fig. 5:**
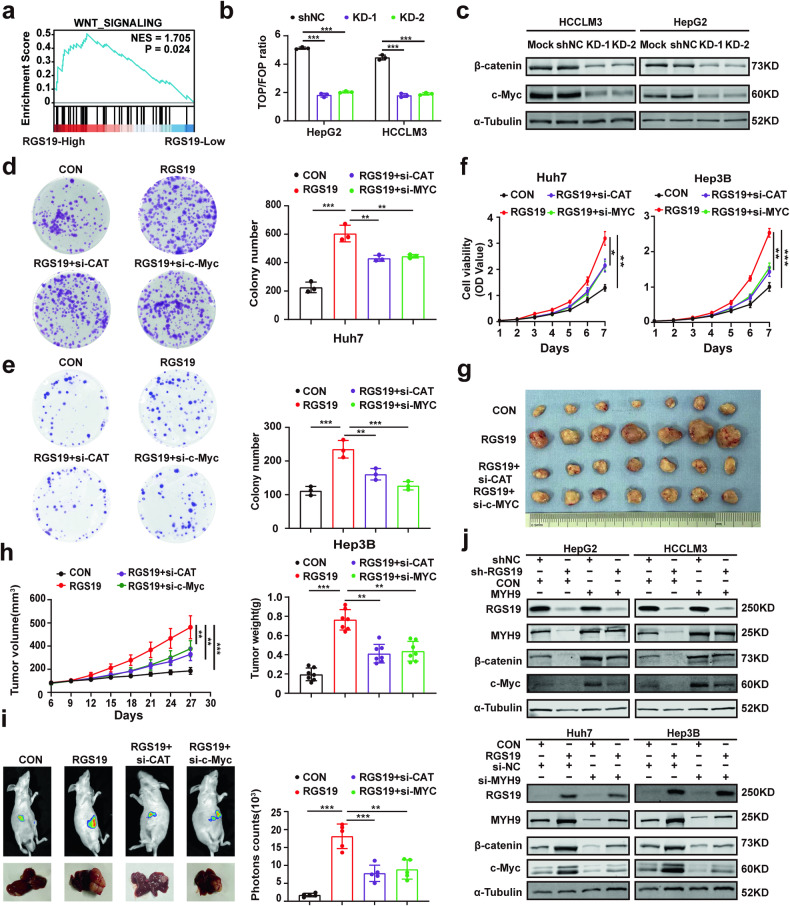
RGS19 activates the β-catenin/c-Myc axis in HCC cells. **a** Gene set enrichment analysis (GSEA) of the TCGA dataset revealed that the genes that were differentially expressed after RGS19 knockdown were significantly enriched in the Wnt signaling pathway. **b** TOP/FOP luciferase reporter assays were employed to detect the transcriptional activity of the Wnt signaling pathway in HCC cells transfected with shRGS19. **c** Western blot analyses of the expression of β-catenin and c-Myc in the indicated HCC cell lines. **d**, **e** Colony formation assays in RGS19-overexpressing cells treated with si-β-catenin or si-MYC in vitro. **f** Analysis of CCK-8 assays in RGS19-overexpressing cells treated with si-β-catenin or si-c-Myc in vitro. **g**, **h** Typical images of subcutaneous xenografts (*n* = 7/group) and analysis of tumor growth and weight. **i** Typical images of the orthotopic liver tumor models (*n* = 7/group) with RGS19-overexpressing cells treated with si-β-catenin or si-c-Myc and analysis of the photon counts of the model. **j** Western blot analysis showed that MYH9 overexpression or knockdown regulated the effect of RGS19 on the expression of β-catenin/c-Myc in HCC cells. All experiments were performed three times, and the data are presented as the mean ± SD. *p < 0.05; **p < 0.01; ***p < 0.001. GSEA: gene set enrichment analysis; siRNA: small interfering RNA.

A previous study reported that MYH9 activates the Wnt/β-catenin signaling pathway by promoting the degradation of GSK-3β in HCC^16^. Moreover, we also found that the Wnt signaling pathway was significantly enriched in RS19-high HCC tissues in a TCGA dataset (Fig. 5a). Therefore, we investigated whether RGS19 regulates the β-catenin/c-Myc axis. First, TCF/LEF reporter assays, which specifically detect the activation of the Wnt pathway, were used to test whether RGS19 regulates the Wnt pathway. The results indicated that knockdown of RGS19 decreased the transcriptional activity of β-catenin, while overexpression of RGS19 had the opposite effect (Fig. 5b and Supplementary Fig. 19a). Next, we found that the protein levels of β-catenin and c-Myc were increased in RGS19-overexpressing HCC cells and decreased in HepG2-shRGS19 and HCCLM3-shRGS19 cells (Fig. 5c and Supplementary Fig. 19b). We also investigated the role of the β-catenin/c-Myc axis in RGS19-mediated HCC proliferation. The results showed that si-β-catenin or si-c-Myc significantly reduced the proliferative ability of HCC cells mediated by RGS19 overexpression, as determined by colony formation assays and CCK-8 assays (Fig. 5d–f). Moreover, both bioluminescence imaging and tumor specimen analysis revealed that tumor volume and weight were decreased by si-β-catenin or si-c-Myc in RGS19-overexpressing cells in vivo (Fig. 5g–i). Finally, we tested whether RGS19 regulates the β-catenin/c-Myc axis via MYH9. The results indicated that transfection with si-MYH9 plasmids significantly suppressed the expression of β-catenin/c-Myc mediated by RGS19 overexpression, while the expression of β-catenin/c-Myc was restored in sh-RGS19 HCC cells transfected with MYH9 plasmids (Fig. 5j).

### c-Myc directly binds to the promoter region of RGS19 and forms a positive feedback loop in HCC

**Fig. 6:**
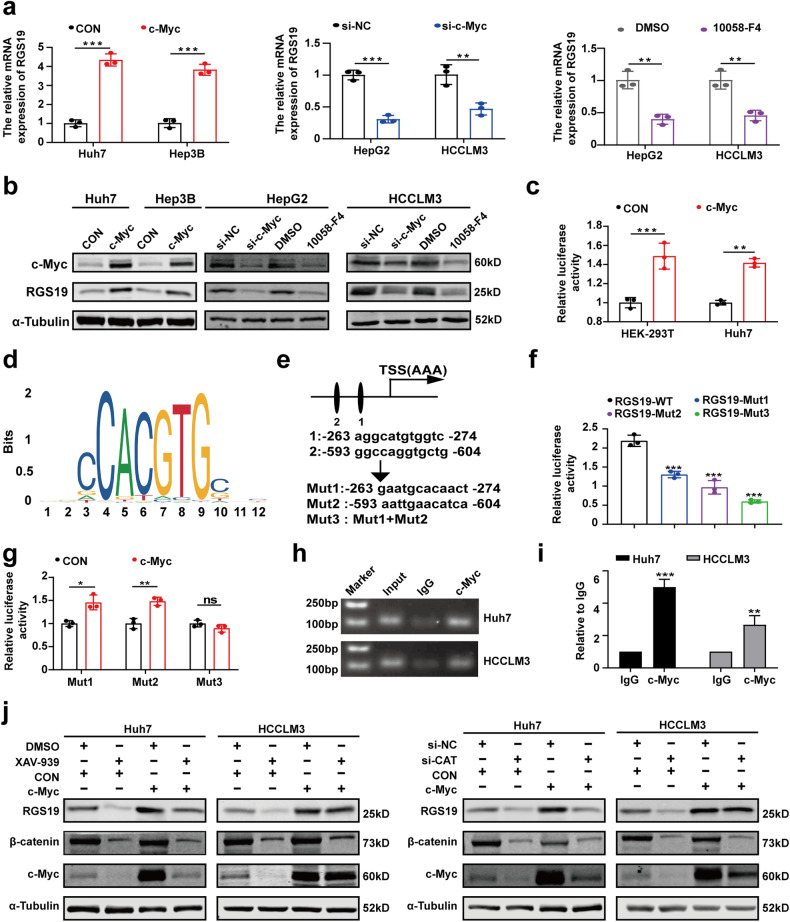
c-Myc binds to the RGS19 promoter and upregulates RGS19 expression. **a**, **b** qRT‒PCR and Western blotting were used to assess the expression of RGS19 in HCC cells treated with the c-Myc plasmid, si-c-Myc, or the c-Myc inhibitor (10058-F4). **c** Luciferase reporter assays were performed in HEK-293T and Huh7 cells co-transfected with RGS19-promoter-luc and c-Myc or the vector plasmid. **d** c-Myc binding motif. **e** Two potential MYC-binding sites within the RGS19 promoter region and three corresponding mutants of the RGS19 promoter. **f** HEK-293T cells were transfected with different luciferase reporter vectors containing mutants of the RGS19 promoter. **g** Luciferase reporter assays were performed in HEK-293T cells co-transfected with RGS19 luciferase reporter vectors (containing mutants of the RGS19 promoter) and c-Myc or vector plasmid. **h** Agarose electrophoresis for ChIP analysis of c-Myc binding to the RGS19 promoter. **i** ChIP‒qPCR assays showed that c-Myc directly binds to the RGS19 promoter region in Huh7 and HCCLM3 cells. **j** Western blot analysis showed that c-Myc overexpression increased the level of RGS19 suppressed by si-β-catenin or XAV-939 in HCC cells. All experiments were performed three times, and the data are presented as the mean ± SD. *p < 0.05; **p < 0.01; ***p < 0.001.

As a canonical transcription factor, c-Myc contributes to cancer progression by transcriptionally activating downstream genes. Therefore, we sought to explore whether c-Myc regulates RGS19 expression in HCC cells. The results demonstrated that the RGS19 mRNA and protein levels were significantly increased by c-Myc overexpression, while the opposite trend was observed after c-Myc knockdown or 10058-F4 (an inhibitor of c-Myc) treatment in HCC cells (Fig. 6a, b). These results indicated that c-Myc could regulate RGS19 expression in HCC cells. Next, we performed luciferase reporter assays and found that the overexpression of c-Myc upregulated RGS19 promoter activity in Huh7 and HEK293T cells (Fig. 6c). Because the promoter of RGS19 contains 2 potential c-Myc binding sites (JASPAR), we aimed to identify which binding sites are necessary for c-Myc. We mutated the putative binding sites separately or together (Fig. 6d, e). The activity of the RGS19 promoter gradually decreased when the binding sites were mutated separately or together (Fig. 6f). We also found that c-Myc did not increase the activity of the RGS19 promoter when both binding sites were mutated (Fig. 6g). These results suggested that both binding sites in the RGS19 promoter are necessary for c-Myc binding. Subsequent ChIP assays in Huh7 and HCCLM3 cells further demonstrated that c-Myc is directly bound to the RGS19 promoter (Fig. 6h, i). Therefore, these results illustrated that there is a mutual regulatory relationship between RGS19 and c-Myc in HCC cells.

Finally, we tested whether RGS19/β-catenin/c-Myc formed a regulatory circuit. We found that the overexpression of c-Myc significantly abrogated the suppressive effect of si-β-catenin or XAV-939 (an inhibitor of β-catenin) on the expression of RGS19 in Huh7 and HCCLM3 cells (Fig. 6j).

### Correlations between RGS19 and MYH9, β-catenin or c-Myc in an HCC animal model and in HCC clinical samples

**Fig. 7:**
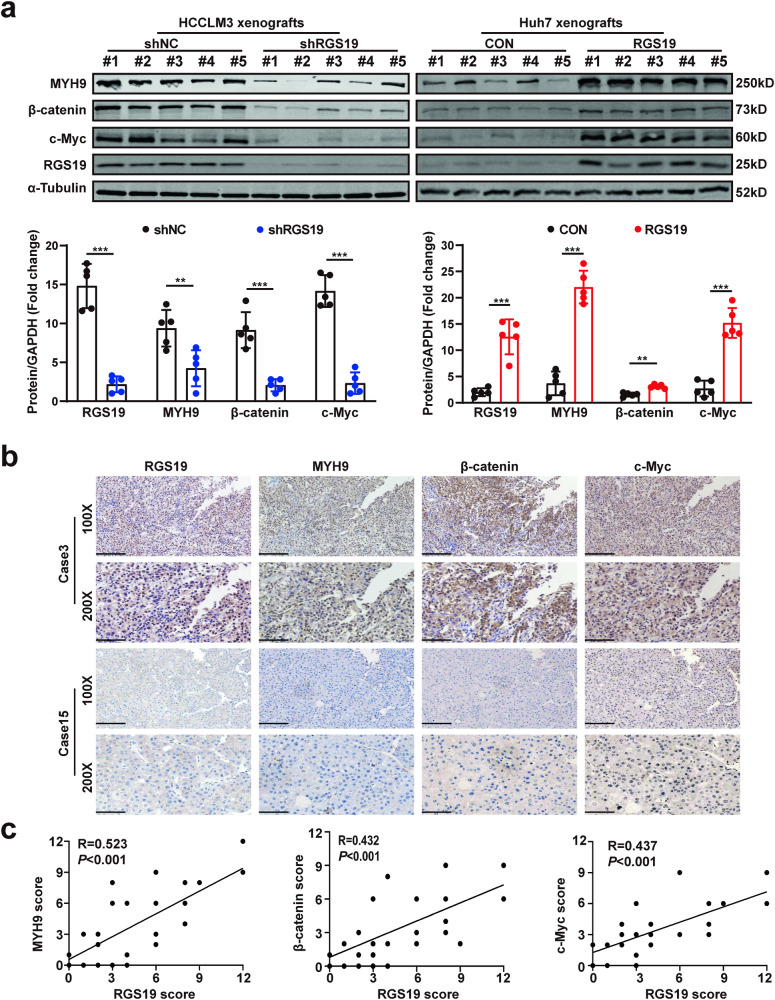
Correlations between RGS19 and MYH9, β-catenin and c-Myc. **a** Western blot analyses of the expression of MYH9, β-catenin, and c-Myc in RGS19-knockdown and RGS19-overexpressing xenograft tissues. **b** Representative images of IHC staining of RGS19, MYH9, β-catenin, and c-Myc in serial sections from HCC tissues (*n* = 30). Scale bars: 100× = 200 μm; 200× = 100 μm. **c** Correlation analysis suggested that RGS19 is positively associated with MYH9, β-catenin and c-Myc. Pearson correlation analysis was used. All experiments were performed three times, and the data are presented as the mean ± SD. *p < 0.05; **p < 0.01; ***p < 0.001.

**Fig. 8:**
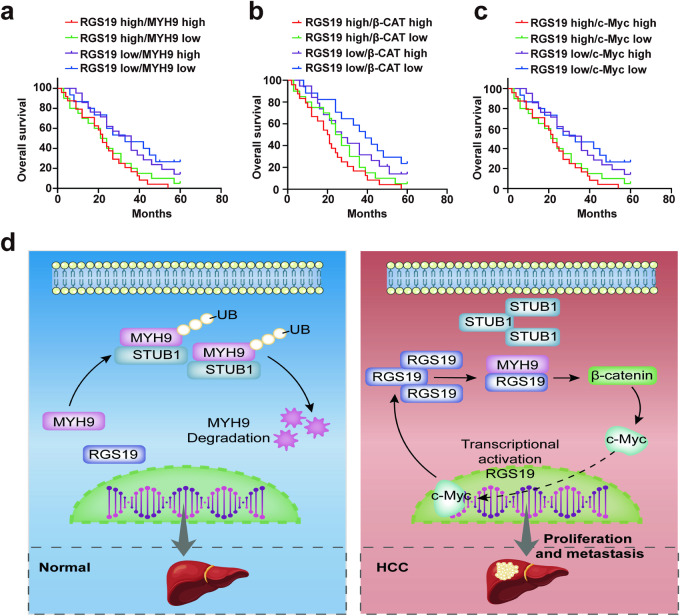
Analysis of the clinical prognosis of HCC patients with different expression levels of RGS19 MYH9, β-catenin and c-Myc. **a** Kaplan‒Meier analysis of OS in patients with variable expression of RGS19 and MYH9. **b** Kaplan‒Meier analysis of OS in patients with variable expression of RGS19 and β-catenin. **c** Kaplan‒Meier analysis of OS in patients with variable expression of RGS19 and c-Myc. **d** Schematic representation of the mechanism by which RGS19 mediates HCC proliferation and metastasis. All experiments were performed three times, and the data are presented as the mean ± SD. *p < 0.05; **p < 0.01; ***p < 0.001. OS: overall survival.

To further confirm the correlations between RGS19 and downstream genes, we first determined their expression in murine xenografts from RGS19-knockdown and RGS19-overexpressing HCC cells, and the trend was similar to what we have mentioned above. The expression of downstream genes was elevated in the xenograft tissues of mice injected with RGS19-overexpressing cells, while the expression of genes was decreased in the tissues of mice injected with RGS19-knockdown cells (Fig. 7a). Next, based on a TCGA dataset, we also found that the expression of RGS19 was positively correlated with the expression of MYH9 (*r* = 0.413, *p* < 0.001), β-catenin (*r* = 0.224, *p* < 0.001) and c-Myc (*r* = 0.187, *p* < 0.001) (Supplementary Fig. 20). Moreover, we determined the expression of RGS19 in serial clinical HCC tissue sections via IHC, and the expression of RGS19 was positively associated with the expression of MYH9 (*r* = 0.523, *p* < 0.001), β-catenin (*r* = 0.432, *p* < 0.001) and c-Myc (*r* = 0.437, *p* < 0.001) (Fig. 7b, c). In addition, we demonstrated that the expression of RGS19 was elevated in high-grade HCC samples (Fig. 1e). Therefore, we explored the expression of MYH9, β-catenin, and c-Myc in HCC samples from different stages. We found that, similar to the trend in the expression of RGS19, the expression of MYH9, β-catenin, and c-Myc was also elevated in high-grade HCC samples (Supplementary Fig. 21). These results confirmed the strong correlations between RGS19 and downstream genes in HCC tissues. Importantly, high expression of both RGS19 and MYH9 or β-catenin was significantly associated with poorer prognosis in HCC patients than in patients in the other groups (Fig. 8a, b). Moreover, patients with simultaneously high expression of RGS19 and c-Myc had worse outcomes (Fig. 8c). These results highlighted that RGS19 combined with MYH9/β-catenin/c-Myc could be an alternative therapeutic target for patients with HCC. In conclusion, RGS19 and its downstream genes formed a positive feedback loop and promoted the development of HCC (Fig. 8d).

## Discussion

HCC is one of the most common life-threatening malignancies because of its subtle onset and high mortality rate. Therefore, identifying not only the underlying mechanisms that contribute to HCC proliferation and metastasis but also novel biomarkers that can improve diagnostic accuracy is urgently needed. In this study, we discovered that upregulated RGS19 expression is significantly associated with tumor size, AFP level, OS, and PFI in patients with HCC. Our results demonstrated that independent of its GAP function, RGS19 enhances the proliferative and metastatic ability of HCC cells by interacting with MYH9. RGS19 increased the protein level of MYH9 by antagonizing the STUB1-MYH9 interaction and decreasing MYH9 degradation mediated by STUB1. Our study further revealed that RGS19 activates the β-catenin/c-Myc nexus in an MYH9-dependent manner. Surprisingly, c-Myc could directly regulate the expression of RGS19 by interacting with the promoter region of RGS19. Overall, we highlighted the positive regulatory loop formed by RGS19/MYH9/β-catenin/c-Myc, which promotes the proliferation of HCC cells.

RGS19, a member of the RGS family, is involved in promoting the termination of the G protein signaling pathway by catalyzing GTPase activity. Previous studies have demonstrated that abnormal expression of RGS proteins is associated with pathological conditions^30–32^. However, the relationship between RGS19 and HCC has not yet been well studied. In this study, we are the first to report that RGS19 is overexpressed in HCC tissues. Elevated RGS19 expression was significantly associated with clinical parameters and poor prognosis in patients with HCC. Further functional experiments also suggested that RGS19 promoted the proliferation and metastasis of HCC cells in vitro and in vivo independent of its GAP functions. These results demonstrated that RGS19 could be an effective prognostic marker and molecular target for HCC therapy via novel biological mechanisms.

Next, we identified MYH9 as a protein that interacts with RGS19 via mass spectrometry. MYH9 is a typical oncogene that promotes cell proliferation, metastasis, stemness, and drug resistance in multiple cancers^33–35^. MYH9 promotes the stemness of HCC cells by activating the Wnt signaling pathway, but how the expression of MYH9 is regulated in HCC remains unclear. In this study, we demonstrated that RGS19, independent of its GAP function, noncatalytically inhibits MYH9 ubiquitination by competing with STUB1 for binding to the same motor domain of MYH9. This direct counter regulation between RGS19 and an E3 ligase might reveal an intricate mechanism in the mammalian ubiquitin signaling system. Previous studies have shown that TRIM26 protects SOX2 from proteasomal degradation by inhibiting the interaction of WWP2 with SOX2^36^. Liu et al. reported that RFC4 directly binds to NICD1 to competitively abrogate CDK8/FBXW7-mediated degradation of NICD1^37^. Zhong et al. showed that ATG9B and MYH9 enhance their stability by suppressing their interaction with STUB1^15^. However, in contrast to the findings above, RGS19 and STUB1, which belong to different protein families, have diametrically opposite functions in regulating MYH9 ubiquitination. Our truncation assays indicated that the RGS domain of RGS19 is necessary for its interaction with MYH9, increasing the protein stability of MYH9. Additionally, our findings suggest that other RGS-containing proteins might bind to and regulate the stability of MYH9. Moreover, RGS19 might regulate other factors through similar mechanisms.

Abnormal activation of the Wnt/β-catenin signaling pathway plays an essential role in the initiation and progression of HCC. Hence, pharmacological inhibitors of Wnt/β-catenin are necessary to improve the therapeutic efficacy of HCC treatments. C-Myc, a downstream target gene of β-catenin, also plays pivotal roles in tumorigenesis in human and mouse models^38,39^. C-Myc performs its oncogenic functions primarily as a transcription factor that activates the expression of genes at the transcriptional level. Lin et al. reported that MYH9 activated the canonical Wnt/β-catenin signaling pathway by interacting with GSK3β and promoting its degradation^16^. Using a TCGA dataset, we found that the expression of RGS19 was significantly associated with the Wnt signaling pathway. TOP/FOP assays indicated that RGS19 regulates the activation of β-catenin. In addition, we found that RGS19 regulates the expression of the β-catenin/c-Myc axis via MYH9. By bioinformatic analysis, we found that the RGS19 promoter contains c-Myc binding sites, and further investigations showed that c-Myc could directly interact with the RGS19 promoter region and transcriptionally activate RGS19 expression. Our study also indicated that the overexpression of c-Myc significantly alleviated the suppressive effect of si-β-catenin or the β-catenin inhibitor XAV-939 on the expression of RGS19. Therefore, a positive feedback loop involving RGS19/MYH9/β-catenin/c-Myc was validated in HCC cells. Although our study illustrated novel and biologically essential roles for RGS19 in regulating MYH9 protein stability and forming a feedback loop with c-Myc, whether RGS19 performs other biological functions through its noncatalytic activity in HCC remains unclear. Hence, further research on the functions of RGS19 in HCC is needed.

In conclusion, we demonstrated that RGS19 is an oncoprotein that promotes the proliferation and invasion of HCC cells. Our study revealed the crucial role of RGS19 in activating the β-catenin/c-Myc axis via MYH9. Furthermore, increased expression of c-Myc also directly increases RGS19 expression. This regulatory circuit formed by RGS19, MYH9, and the β-catenin/c-Myc axis synergistically promoted the development of HCC. These findings further deepen our understanding of the role of RGS19 in HCC. Consequently, RGS19 could be an effective molecular target and prognostic biomarker for HCC.

### Supplementary information

Supplementary material

## Data Availability

All the data generated and analyzed during this study are included in this article and its additional file.
