# Supplementary material for: RGS19 activates the MYH9/β-catenin/c-Myc positive feedback loop in hepatocellular carcinoma
Source: Exp Mol Med. 2024 Jun 3;56(6):1412–25. doi: 10.1038/s12276-024-01244-9 (PMC11263569; doi:10.1038/s12276-024-01244-9)
Supplement: Supplementary file 1 — Supplementary material [file 12276_2024_1244_MOESM1_ESM.pdf]

Supplementary Material

Materials and Methods

RNA isolation and quantitative real-time PCR

Total RNA from tissues and cells was extracted using an RNA Miniprep Kit (Axygen, China) and quantified with a Nanodrop ND-2000 (Thermo, USA). The cDNA was synthesized with a Reverse Transcription Kit (Applied Biosystems). Real-time PCR was performed using SYBR Green (Roche, USA) on an ABIPRISM 7500HT instrument (Applied Biosystems). The expression of mRNA was normalized to that of GAPDH. The primer sequences are shown in Supplementary Table 2

Wound-healing assay

A total of 30×10^4 transfected cells were seeded into six-well plates and cultured until cells were confluent. The bottom of each well was straightly scratched with a 10 μL pipette tip, each well was washed with PBS, and the cells were cultured with serum-free medium for 24 h. The wound-healing results were observed at 0 and 24 h with a microscopy and camera.

Migration and invasion assay

For transwell-migration assays, transfected cells in serum-free medium were plated in the upper chamber of the Transwell system. Normal medium was added into the 24-well plate. During the invasion assays, Matrigel-coated (BD Biosciences, San Joe, CA, USA) transwell chambers were used to examine the invasion ability of cells. The following procedures were similar to those used for the migration assays. After a 24-48

h incubation time, the cells on the bottom surface of the filter membranes were fixed, stained and counted under a light microscope.

Cell proliferation and colony formation assay

For cell counting kit-8 (CCK-8) assays, transfected cell suspensions were seeded in 96-well plates (1×10^3 cells/well). CCK-8 solution (Dojindo, Japan) was added to each well at the indicated time points. After incubation for two hours at 37 °C, the absorbance at 450 nm was measured. For colony formation assays, 1×10^3 transfected cells were plated into 6-well plates. After incubating for 2 weeks, the culture medium was removed and the colonies were fixed with 4% paraformaldehyde (PFA) and stained with 0.5% crystal violet.

EdU assay

An EdU analysis kit (Beyotime, China) was used to perform EdU dye assays for cells according to the manufacturer’s instructions. Then a fluorescence microscope (Nikon, Japan) was used to detect labelled cells, and the results were evaluated by Image-Pro Plus 6.0 software (Media Cybernetics, Inc., MD, USA). The results were calculated by the following process: percentage of proliferative cells (%) = the number of labelled cells/ the total cell number.

Terminal dUTP nick-end labeling (TUNEL) assay

We performed a TUNEL assay (Roche, Shanghai, China) according to the manufacturer’s instructions to detect apoptosis levels in vivo and quantified TUNEL-positive cells under microscopy.

Flow cytometric analysis

For apoptosis analysis, we used the Annexin V-FITC Apoptosis Detection Kit (Beyotime, China). Briefly, the indicated cells were suspended in 195μl of binding buffer, and 5μl Annexin V-FITC and 10μl of PI were then added. The cells were incubated on the ice in the dark. The flow cytometric analysis was used to detect apoptotic cells.

Lentiviral construction and cell transfection

To generate stable RGS19-overexpressing and RGS19-knockdown cell lines, lentiviral vectors for human RGS19 regulation (Lv-RGS19, Lv-shRGS19) and the corresponding control vectors (Lv-NC) were obtained from HanBio (Shanghai, China). We also used Lipo2000 to transfect siRNA or plasmids targeting MYH9, β-catenin and c-Myc into HCC cell lines.

Immunohistochemical (IHC) staining

The IHC staining procedures were similar to what we previously reported[1]. The tissue sections were incubated with primary antibodies overnight. The next day, the sections were incubated with secondary antibodies (Vector Laboratories, Burlingame, CA, USA). Finally, tissue sections were stained with a diaminobenzidine (DAB) kit (Vector Laboratories) and counterstained with haematoxylin (Sigma-Aldrich, USA). The primary antibodies for IHC included RGS19, MYH9, β-catenin, Ki-67 and c-Myc. The results were based on the intensity and proportion of staining. The staining intensity was scored as 0 (negative), 1 (weak), 2 (moderate), and 3 (strong). The positively stained cell proportion was defined as follows: 0 (negative), 1 (0.01-25%), 2 (25.01-50%), 3 (50.01-75%) and 4 (75.01-100%). The histologic score (H score) was

calculated by multiplying the intensity score and proportion score. Tissue sections with an H score ≤4 were classified into the low expression group, and those with an H score >4 were classified into the high expression group.

Western blot

Tissues and cells were lysed with RIPA buffer containing protease and phosphatase inhibitors. The protein samples were separated on SDS-PAGE gels and transferred onto nitrocellulose membranes. Then, the membranes were incubated with primary antibodies overnight at 4 °C, probed with IRDye 800CW secondary antibodies (LI-COR, USA) and visualized by the Odyssey® Imaging System (LI-COR, USA). Information of the primary antibodies is supplied in Supplementary Table 3.

Co-immunoprecipitation and Mass spectrum (MS)

Cells were lysed in IP lysis buffer (Beyotime, China), incubated for 30 minutes on ice and centrifuged at 12000×g for 15 minutes. Cell lysates were immunoprecipitated with antibodies against Flag and MYH9 or negative control IgG on a rotator at 4 °C overnight. The next day, the A/G plus agarose beads (Santa Cruze, USA) were added into precipitate the protein complex at 4 °C for 4h. Subsequently, samples were washed five times with IP buffer, and the beads were boiled in loading buffer. The protein was prepared for western blot or LC-MS/MS from Aksomics (Shanghai, China). The results of LC-MS/MS were shown in Supplementary Table 4.

CHX assay

The indicated cells were treated with CHX (20μM). These cells were collected at indicated time point and prepared for western blot analysis. Cells were transfected with

scramble control, siRNA or plasmid, and cultured with MG132 (10μM) or CQ (10μM) for 6h. Then the cells were collected and prepared for western blot analysis.

In vitro ubiquitination assay

Cells were transfected with plasmids and siRNAs as indicated, with HA-Ub. After 48h of transfection, the cells were treated with MG132 (100nM) for 24h. The cell lysates prepared with IP lysis buffer (Beyotime, China) were used to immunoprecipitation for MYH9 proteins. The level of MYH9 ubiquitination were detected by anti-Ub or anti-HA antibody.

Immunofluorescent (IF) staining

The detailed procedures of IF have been previously described[2]. Briefly, cells were seeded in confocal dishes. After adherence to the dish, the cells were fixed with 4% paraformaldehyde and permeabilized with Triton X-100. Then, the cells were incubated with specific antibodies and counterstained with 4,6-diamidino-2-phenylindole (Vector, Laboratories). Confocal microscopy (Zeiss LSM 880) was used to take images.

Plasmid construction

RGS19^{Flag}, Ub^{HA} and c-Myc^{Flag} plasmids constructed with the pcDNA3.1(+) vector and MYH9^{His} plasmid constructed with the CMV-M14 vector were obtained from GeneChem (Shanghai, China). P1, P2, P3 truncations of RGS19, SH3-like, Motor, Tail truncations of MYH9 and the full length of STUB1 with its truncations were synthesized by Sangon (Shanghai, China).

Glutathione S-transferase (GST)-pulldown assay

Bacteria-expressed GST or GST-RGS19 and GST-MYH9^{Motro} proteins were purified via GST-tag purification kit (Beyotime, China) following the manufacturer's protocols. His or His-tagged STUB1 and RGS19 were harvested via His-tag purification kit (Beyotime, China). The GST-RGS19 fusion protein was added to cell lysates of HEK-293T cells transfected with MYH9^{His} at 4 °C for 4h. Bound proteins were then analyzed via western blotting.

TOP/ FOP flash reporter assay

The TOP/ FOP-flash reporter and pTK-RL plasmids were co-transfected into cells. After 48h transfection, the Dual-Luciferase Assay Kit (Promega) was utilized according to the instructions. We detected the activities of the both firefly and Renilla luciferase reporters. The TOP-Flash and FOP-Flash reporter activity levels were calculated as the relative ratio of firefly luciferase activity to Renilla luciferase activity.

Luciferase assay

To investigate the regulatory effect of c-Myc on the transcriptional expression of RGS19, the full-length promoter region of RGS19 or mutated c-Myc binding sites of the RGS19 promoter were cloned into the luciferase reporter vector. Then, the WT-RGS19 promoter or MUT-RGS19 promoter reporter plasmids were co-transfected with con or c-Myc plasmids into HEK-293T cells. After transfection for 48h, the cells were harvested, and The Dual-Luciferase Reporter Assay system (Promega) was used to detect luciferase activity.

Chromatin immunoprecipitation assay (ChIP)

A ChIP assay kit was purchased from Millipore. Briefly, HCC cells were crosslinked with 1% formaldehyde, and the cell lysates were sonicated into 500bp fragments. The soluble material was then purified by centrifugation and mixed with a rabbit anti-c-Myc antibody or control rabbit IgG for immunoprecipitation. After that, the magnetic protein A/G beads were added, and the lysis were reverse rotation at 4°C for 24h. The recovered DNA fragments were used to detect the c-Myc binding sites by qPCR. The primers used for ChIP are listed in Supplementary Table 3.

Transcriptomics data analysis

Based on data from TCGA, mRNA expression profiles of HCC patients were applied in correlation analysis, differential expression analysis, survival analysis and Gene Set Enrichment Analysis (GSEA).

Hydrodynamic tail vein injection

Detailed methods have previously described[3,4]. Plasmid dissolved in 2 ml saline was injected into 6-week-old male C57BL/6 mice via the tail vein within 5-7 seconds. We prepared 13 μg of c-Myc, 10 μg of sgP53, 8.25 μg of Sleeping Beauty (SB) transposase and 10 μg of sgRGS19 or empty vector. Vectors for this experiment were prepared using the EndoFreeMaxi kit (Qiagen). The survival of mice and HCC incidence were analyzed.

***In Vivo* Efficacy Studies**

Four- to six- week-old male BALB/C nude mice were perchased from Vital River Laboratory (Beijing, China) and were housed under specific pathogen-free conditions. We performed animal experiments that were approved by the Animal Ethics Committee.

For the subcutaneous xenograft model, 3 × 106 transfected cells suspended in 150 μL PBS were injected into the flanks of the mice. Tumor size was measured weekly. After six weeks, the tumor was removed and its weight and volume were recorded. To establish the liver orthotopic xenograft implantation model, subcutaneous tumours were divided into 1 mm3 sections and were implanted into the liver. The growth of tumours was measured by bioluminescent signals once a week and six weeks later the mice were sacrificed. The pulmonary metastatic model was established by injecting 2 × 106 transfected cells into the tail vein of the mouse. The monitoring of metastatic nodules was achieved by bioluminescent signals. The mouse was sacrificed six weeks later.

Reference

1 Wang, C. *et al.* LncRNA Hnf4αos exacerbates liver ischemia/reperfusion injury in mice via Hnf4αos/Hnf4α duplex-mediated PGC1α suppression. *Redox biology* **57**, 102498, doi:10.1016/j.redox.2022.102498 (2022).

2 Lu, S. *et al.* NNMT promotes the progression of intrahepatic cholangiocarcinoma by regulating aerobic glycolysis via the EGFR-STAT3 axis. *Oncogenesis* **11**, 39, doi:10.1038/s41389-022-00415-5 (2022).

3 Ruiz de Galarreta, M. *et al.* β-Catenin Activation Promotes Immune Escape and Resistance to Anti-PD-1 Therapy in Hepatocellular Carcinoma. *Cancer Discov* **9**, 1124-1141, doi:10.1158/2159-8290.Cd-19-0074 (2019).

4 Xue, W. *et al.* CRISPR-mediated direct mutation of cancer genes in the mouse liver. *Nature* **514**, 380-384, doi:10.1038/nature13589 (2014).

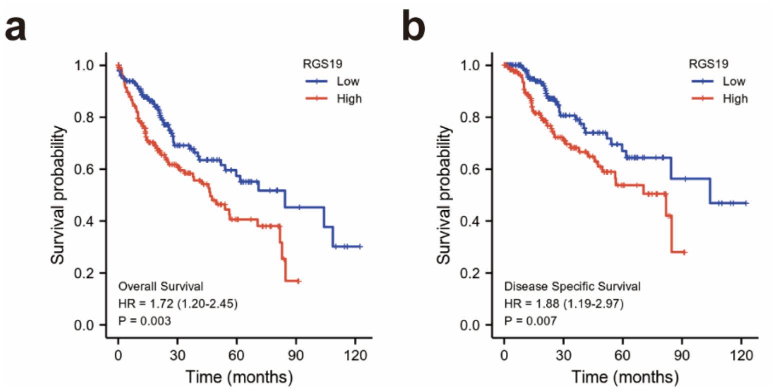

Supplementary Fig. 1.

(a) Patients from the TCGA database with high expression level of RGS19 had poorer overall survival and (b) shorter disease-specific survival than patients with low expression.

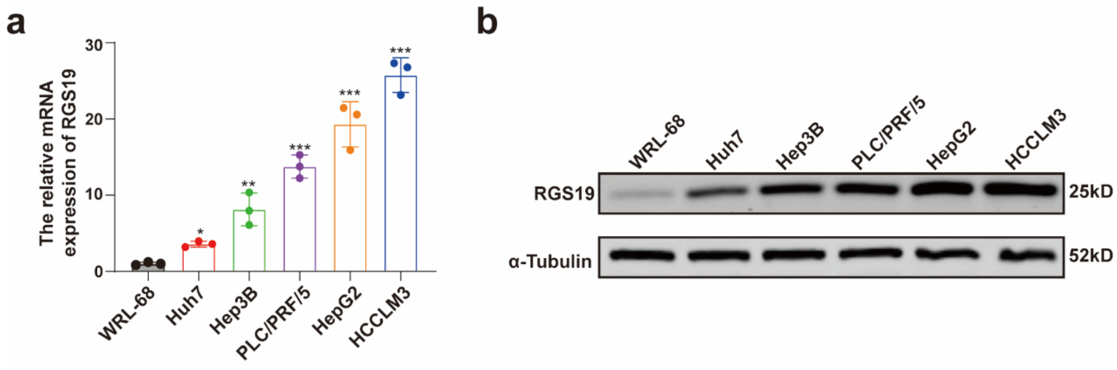

Supplementary Fig. 2.

(a) The expression of RGS19 in HCC cell lines was measured by qRT-PCR and (b) Western blot.

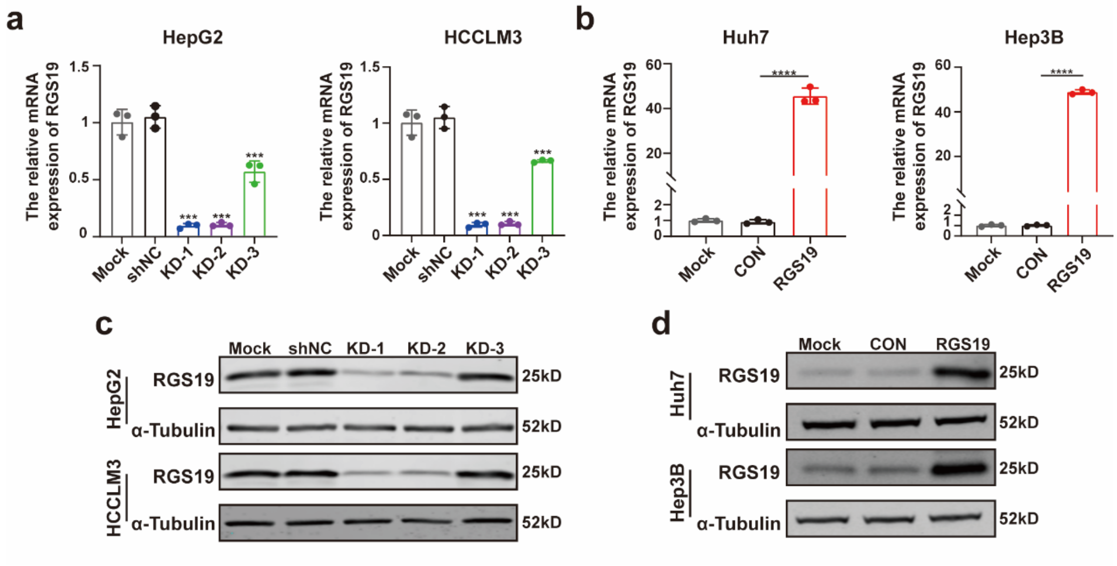

Supplementary Fig. 3.

(a, b) The mRNA expression of RGS19 was detected by q-PCR in HCC cells after lentiviral transfection. (c, d) Western blot analysis of the expression of RGS19 in HCC cells after lentiviral transfection.

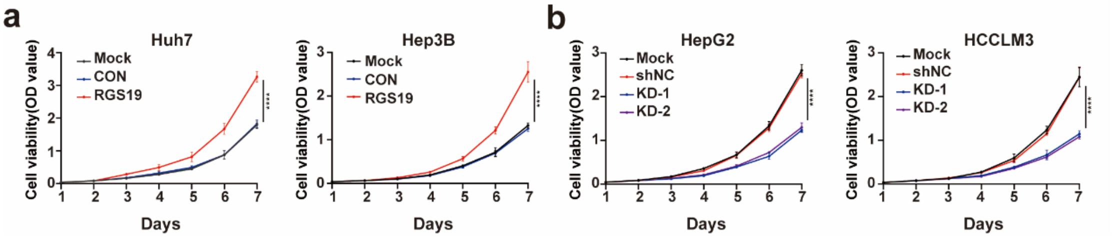

Supplementary Fig. 4.

(a, b) The influence of RGS19 overexpression and knockdown on HCC cells proliferation was evaluated by CCK-8 assays.

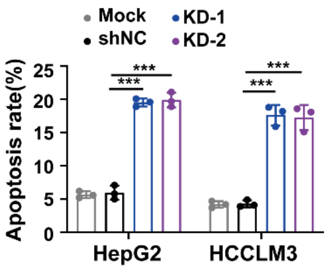

Supplementary Fig. 5.

Analysis of the apoptosis rate mediated by RGS19-knockdown in HCC cells.

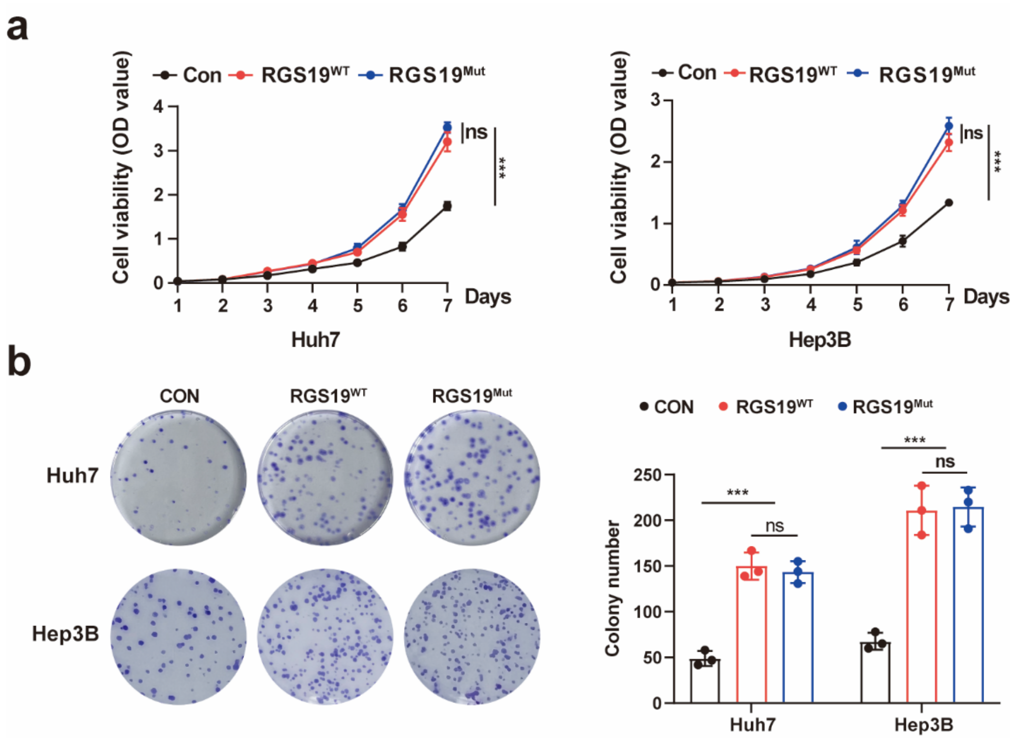

Supplementary Fig. 6.

(a, b) Analysis of CCK-8 assays and colony formation assays in HCC cells transfected with CON, $RGS19^{WT}$ or $RGS19^{Mut}$ plasmids.

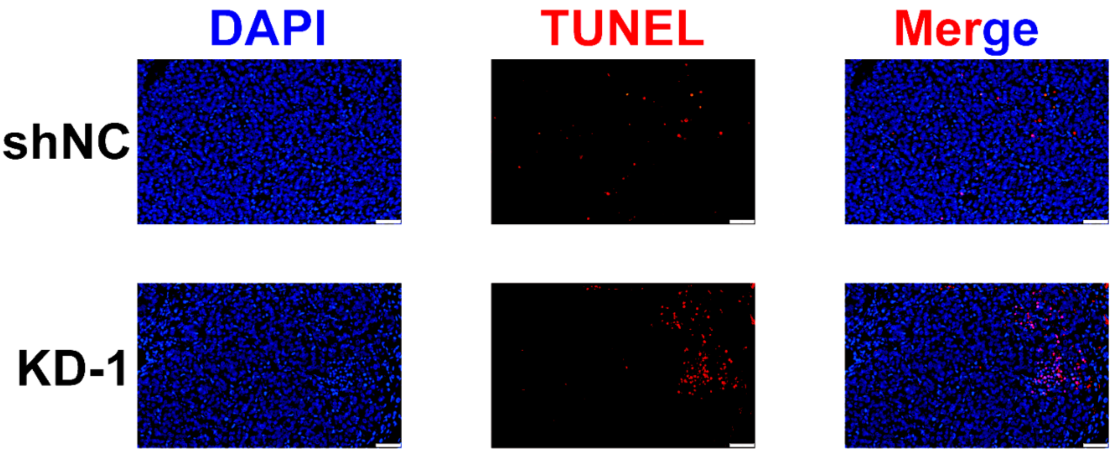

Supplementary Fig. 7.

Analysis of TUNEL positive cells in subcutaneous xenografts tumor sections. Scale bars: 100×= 200 μm.

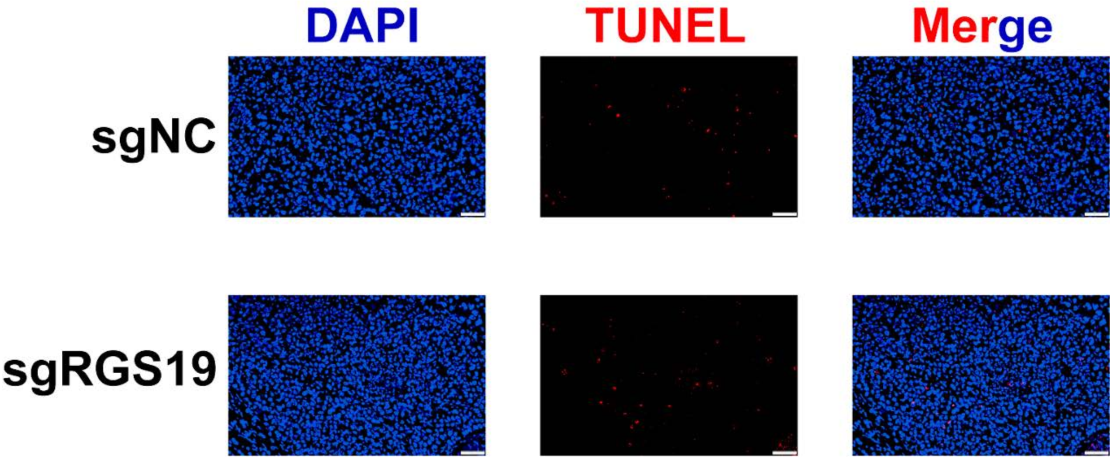

Supplementary Fig. 8.

Analysis of TUNEL positive cells in RGS19-knockout and sgNC tumor sections. Scale bars: 100×= 200 μm.

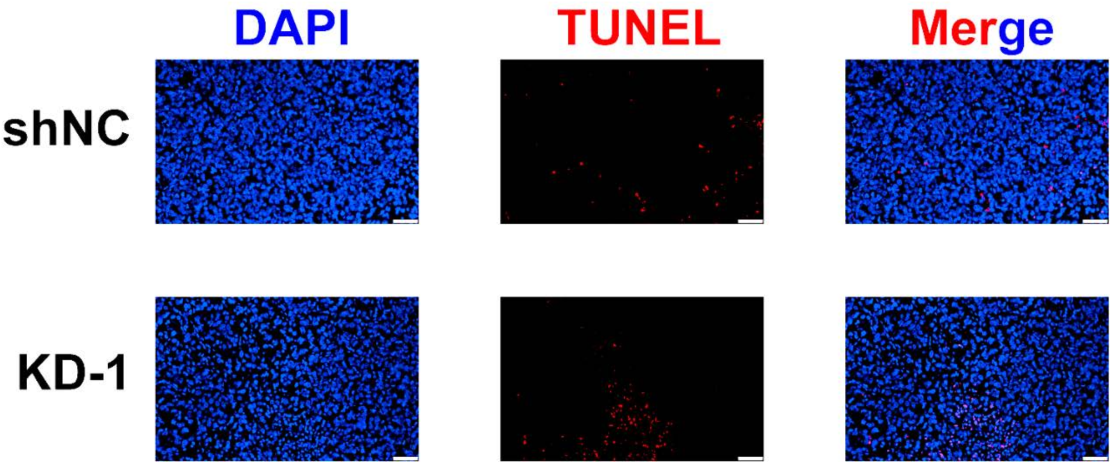

Supplementary Fig. 9.

Analysis of TUNEL positive cells in KD-1 and shNC liver orthotopic tumor sections.

Scale bars: 100×= 200 μm.

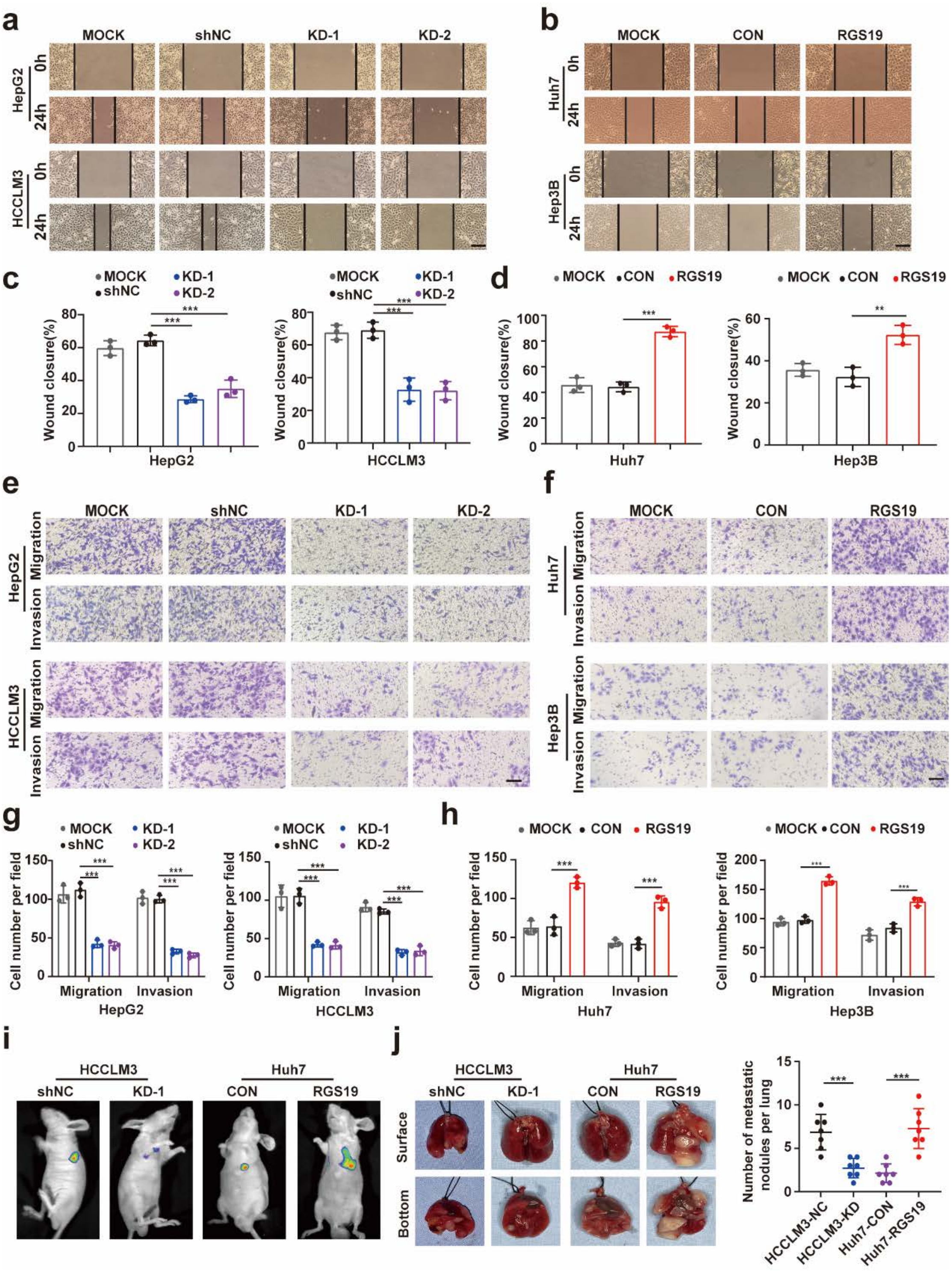

Supplementary Fig. 10.

RGS19 promotes the migration and invasion of HCC cells in vitro and in vivo

(a, b) Representative images of the Wound-healing assay with the indicated HCC cells. Scale bars: 100 μm. (c, d) Statistical analysis of Wound-healing assays. (e, f)

Representative images of Transwell migration and invasion assays with the indicated HCC cells. Scale bars: 100 μm. (g, h) Statistical analysis of the Transwell assays. (i) Representative images of bioluminescence of lung metastasis model from the indicated HCC cells. (j) Lung metastasis specimens and Statistical analysis of the number of metastatic nodules (n=7/group). All experiments were performed three times, and the data were presented as the means ±SD. *p<0.05; **p<0.01; ***p<0.001.

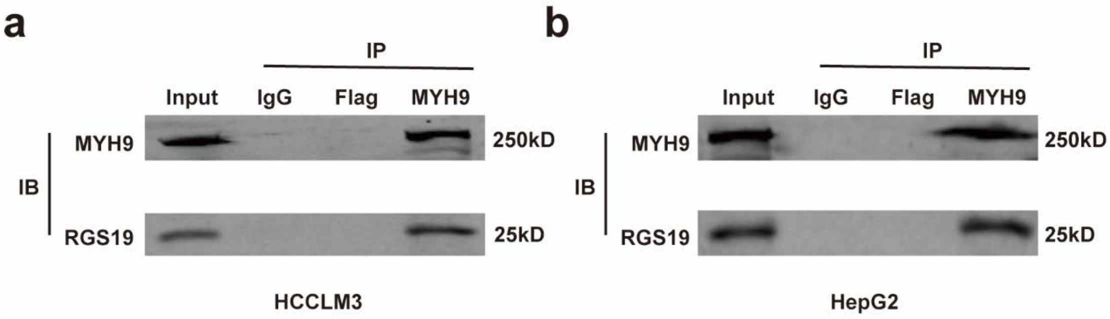

Supplementary Fig. 11.

(a, b) The endogenous interaction between MYH9 and RGS19.

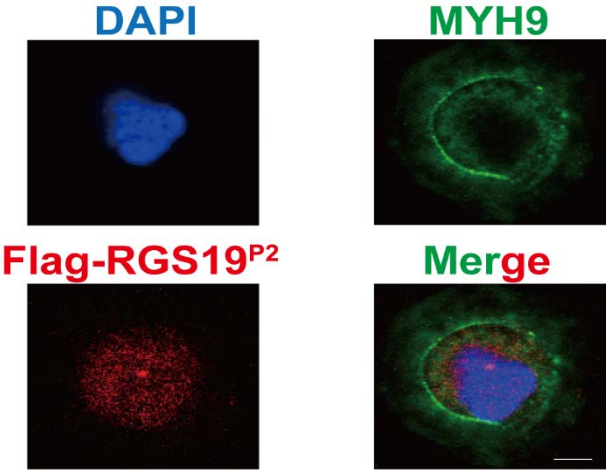

Supplementary Fig. 12.

Typical pictures of co-localization assays between MYH9 and Flag-RGS19^{P2} in Huh7 cells. Scale bars: 10 μm.

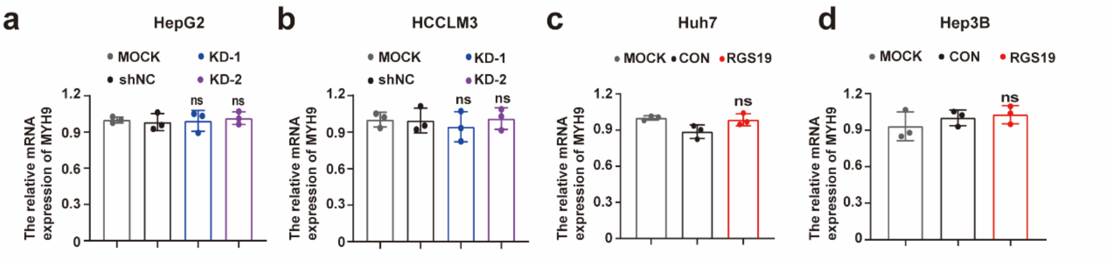

Supplementary Fig. 13.

(a-d) MYH9 mRNA levels in RGS19-overexpressing and RGS19-knockdown HCC cells were measured by qRT-PCR.

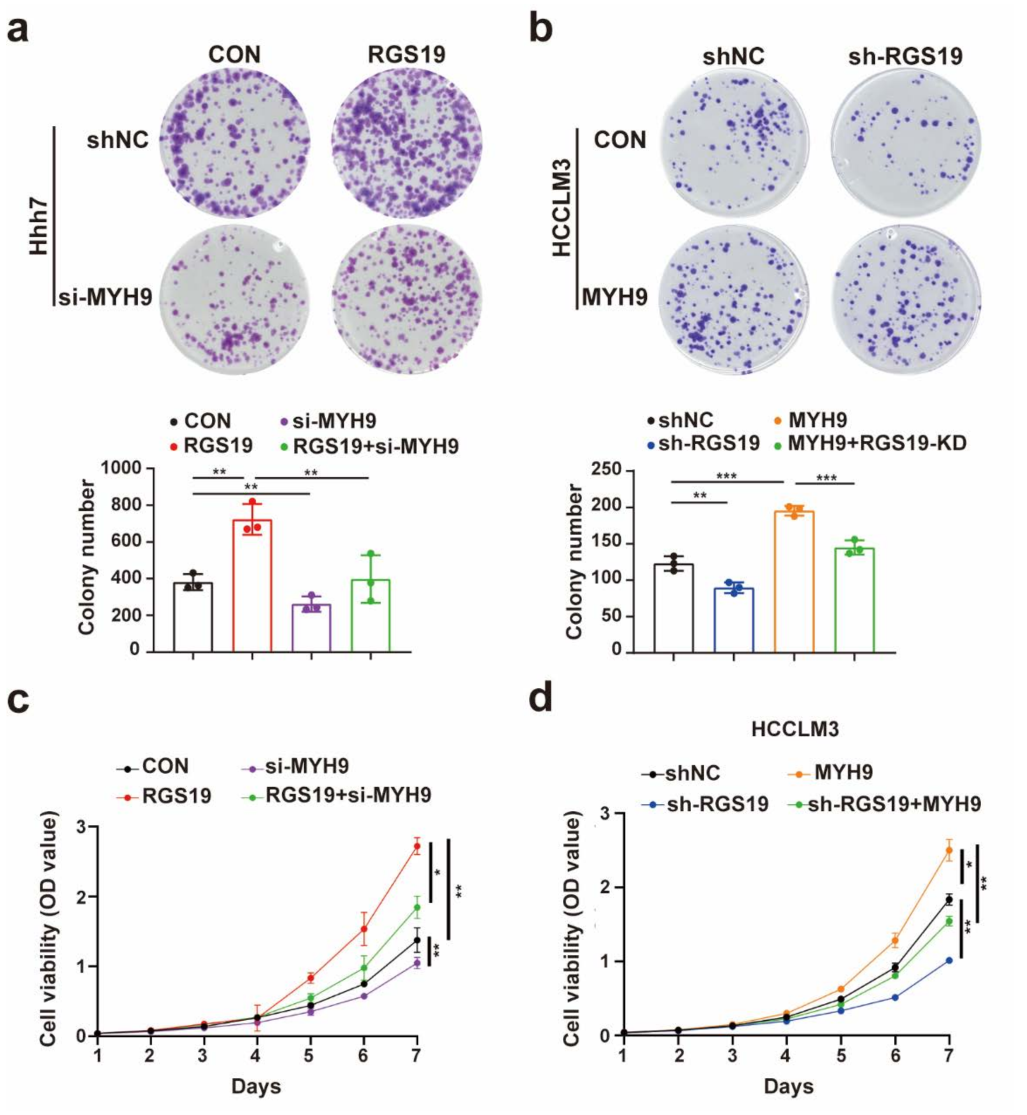

Supplementary Fig. 14.

(a, b) Typical images and analysis of colony formation assays after MYH9 silencing or upregulation in Huh7-RGS19 and HCCLM3-shRGS19 cells. (c, d) The CCK-8 assays were performed after MYH9 silencing or upregulation in Huh7-RGS19 and HCCLM3-shRGS19 cells.

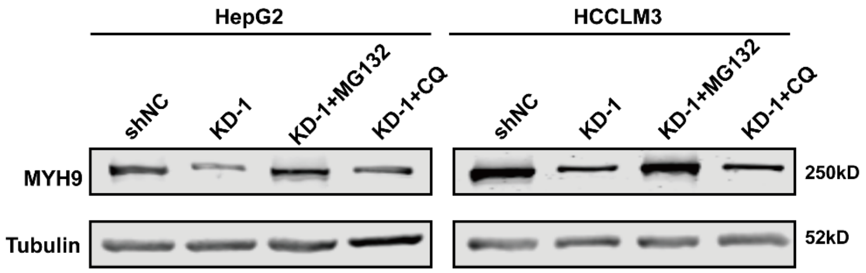

Supplementary Fig. 15.

The expression of MYH9 was detected by Western blot

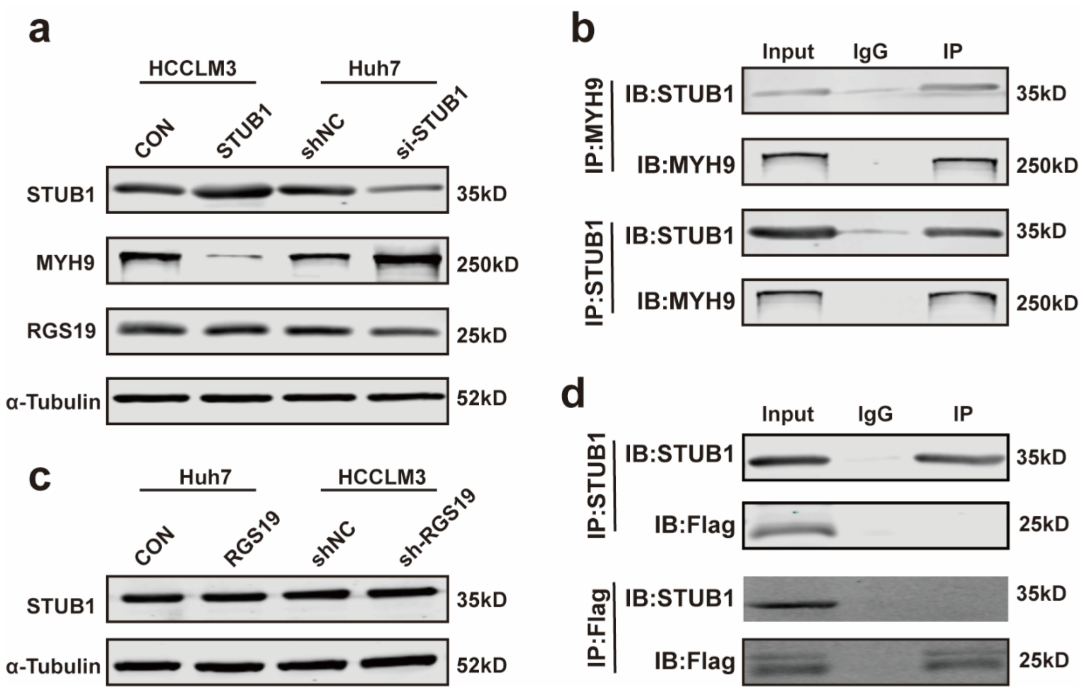

Supplementary Fig. 16.

(a) Western blot analysis the expression of MYH9 and RGS19 after STUB1 upregulation and silencing. (b) Co-IP assays analysis of the interaction between MYH9 and STUB1 in HCC cells. (c) Western blot analysis the expression of STUB1 after RGS19 overexpressing and silencing. (d) Co-IP assays analysis of the interaction between RGS19 and STUB1 in HCC cells.

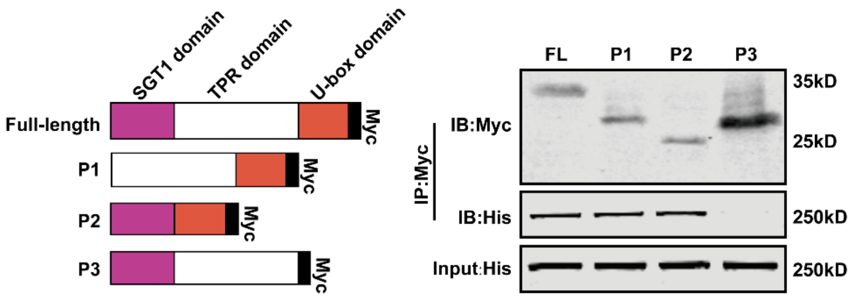

Supplementary Fig. 17.

Full-length of STUB1^{Myc} and its truncated forms (P1, P2 and P3) with MYH9^{His} were co-transfected into HEK-293T cells. Cell lysates of 293T cells were immunoprecipitated with anti-Myc (against STUB1). Immunoblot analysis was performed with anti-His (against MYH9).

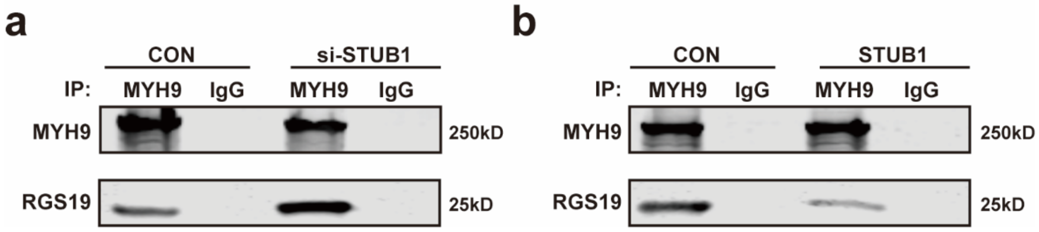

Supplementary Fig. 18.

(a) Co-IP assays analysis of the interaction of MYH9 and RGS19 after STUB1 silencing in Huh7 cells. (b) Co-IP assays analysis of the interaction between MYH9 and RGS19 after STUB1 overexpression in HCCLM3 cells.

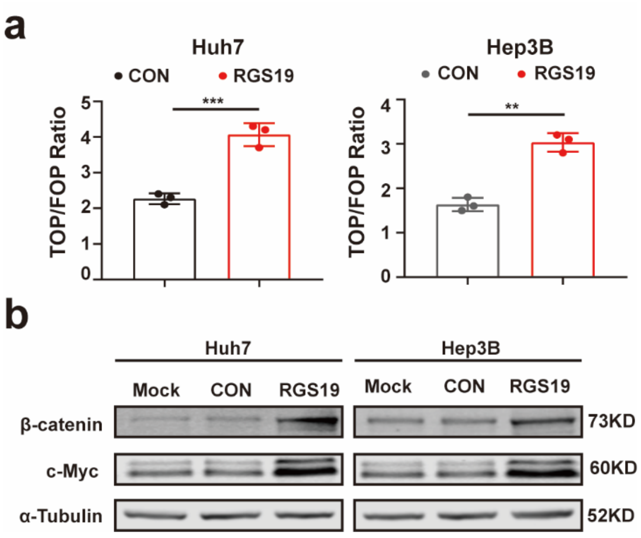

Supplementary Fig. 19.

(a) TOP/ FOP luciferase reporter assays were used to detect the transcriptional activity of Wnt signaling pathway in HCC cells with RGS19 overexpression. (b) Western blotting indicated the expression of β-catenin and c-Myc in HCC cells with RGS19 overexpression.

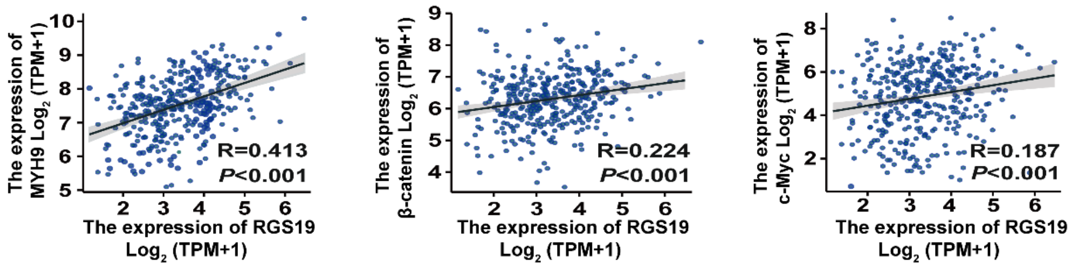

Supplementary Fig. 20.

The correlations between RGS19 and MYH9, β-catenin or c-Myc in TCGA database.

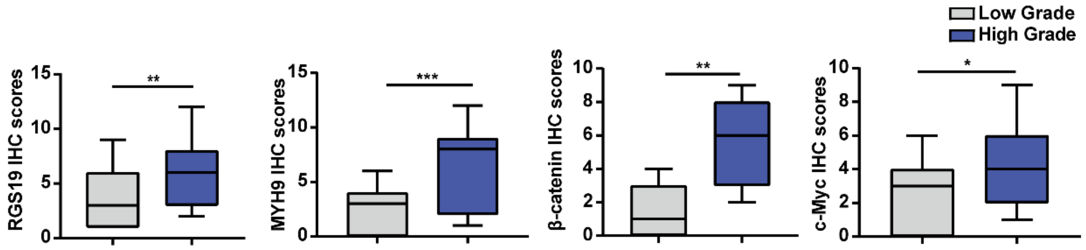

Supplementary Fig. 21.

The IHC scores of RGS19, MYH9, β-catenin and c-Myc in low grade and high grade HCC tissues.

Supplementary Table 1.

Correlation between the clinicopathologic parameters and the expression of RGS19 in HCC.

Characteristic	Low expression of RGS19 (n=47)	High expression of RGS19 (n=50)	p
T stage, n (%)			0.014
T1&T2	26 (26.8%)	15 (15.4%)	
T3&T4	21 (21.6%)	35 (36.1%)	
Tumor diameter, n (%)			0.014
≤5	32 (33%)	21 (21.6%)	
>5	15 (13.4%)	29 (32%)	
HBV infection, n (%)			0.409
Yes	30 (30.9%)	27 (27.8%)	
No	17 (17.5%)	23 (23.7%)	
Gender, n (%)			0.083
Female	27 (27.8%)	38 (39.2%)	
Male	20 (20.6%)	12 (12.4%)	
Age, n (%)			0.31
<=60	24 (24.7%)	31 (31.9%)	
>60	23 (23.7%)	19 (19.6%)	

Characteristic	Low expression of RGS19 (n=47)	High expression of RGS19 (n=50)	p
Histologic grade, n (%)			0.041
G1&G2	31 (32.0%)	22 (22.6%)	
G3&G4	16 (16.5%)	28 (28.9%)	
AFP(ng/ml), n (%)			0.003
<=400	36 (37.1%)	23 (23.7%)	
>400	11 (11.3%)	27 (27.8%)	
Vascular invasion, n (%)			0.419
No	25 (25.8%)	22 (22.7%)	
Yes	22 (22.7%)	28 (28.9%)	

Supplementary Table 2.

The primers used in this study

Gene	Type	Sequence (5'-3')
RGS19	FP	GGCGCAGTCTTTTGACAAGC
	RP	GCCTTCTCGTCTACCACATGC
MYH9	FP	AGTTTGTCTCGGAGCTGTGG
	RP	GGTTCGTGTTCCTCAGCGTA
c-Myc	FP	GTCAAGAGGCGAACACACAAC
	RP	TTGGACGGACAGGATGTATGC
Promoter of RGS19-1	FP	AGGCATGTGGTCCAAAG
	RP	GAGCTGGGCAGTTGATG
Promoter of RGS19-2	FP	GGTGCTGGATGTGCAAAG
	RP	GACCACATGCCTTCCTTC
Promoter of RGS19-2	FP	GACGCCTGCCTGTAGAAAG
	RP	GGGATTTGAACCCAGGACC
GAPDH	FP	GGAGCGAGATCCCTCCAAAAT
	RP	GGCTGTTGTCATACTTCTCATGG

Supplementary Table 3.

The primary antibodies used in this study

Antibody	Host	Product number	Company	Dilution
RGS19	Rabbit	NBP2-24516	Novus	1:1000(WB)
				1:100(IHC)
MYH9	Rabbit	11128-1-AP	Protentech	1:1000(WB)
				1:100(IF)
				1:200(IHC)
				1:50(IP)
FLAG	Rabbit	80010-1-RR	Protentech	1:1000(WB)
				1:100(IF)
				1:50(IP)
His	Mouse	66005-1-Ig	Proteintech	1:1000(WB)
				1:100(IP)
Myc	Rabbit	16286-1-AP	Proteintech	1:1000(WB)
				1:100(IP)
HA	Rabbit	51064-2-AP	Proteintech	1:1000(WB)
Ubiquitin	Mouse	Sc-8017	Santa Cruz	1:100(WB)
Beta-catenin	Rabbit	#8480	Cell Signaling Technology	1:1000(WB)
				1:100(IHC)
C-Myc	Rabbit	#9402	Cell Signaling Technology	1:1000(WB)
				1:50(ChIP)
C-Myc	Rabbit	ab32072	Abcam	1:50(IHC)
Ki-67	Rabbit	Ab16667	Abcam	1:200(IHC)
GAPDH	Mouse	60004	Proteintech	1:10000(WB)
α-Tubulin	Mouse	66031	Proteintech	1:10000(WB)
STUB1	Rabbit	ab134064	Abcam	1:1000(WB)
				1:100(IP)

Supplementary Table 4.

The results of LC-MS/MS

Symbol	IGG.1	Test.1	Test	IGG	fc..Test--IGG	iBAQ
MYH9	0	331	331	0	7.567928	56156000
TUBA1B	0	99	99	0	5.844306	1.98E+08
TUBB4B	0	81	81	0	5.560369	93534000
EEF1A1	0	78	78	0	5.507097	99350000
ACTB	0	74	74	0	5.432861	1.47E+08
PRKDC	0	62	62	0	5.184054	1712500
MYH10	0	57	57	0	5.066225	3053300
FLNA	0	57	57	0	5.066225	2297800
VIM	0	52	52	0	4.937909	21581000
HSP90AB1	0	40	40	0	4.573449	11900000
RPS3	0	39	39	0	4.538476	34496000
SPTBN1	0	37	37	0	4.465879	1335100
SLC25A6	0	34	34	0	4.349637	62734000
HSPA8	0	34	34	0	4.349637	8529200
RPS8	0	33	33	0	4.308711	35259000
RPL6	0	32	32	0	4.26659	42819000
ATP5A1	0	31	31	0	4.223202	13260000
PKM	0	30	30	0	4.178469	10123000
P67936-2	0	29	29	0	4.132304	34795000
SRSF1	0	29	29	0	4.132304	10188000
SPTAN1	0	28	28	0	4.084613	1179900
RPL8	0	26	26	0	3.984224	53894000
SERPINH1	0	26	26	0	3.984224	11964000
GNB2L1	0	25	25	0	3.931283	4825800
MYL6	0	24	24	0	3.876324	82051000
RPL4	0	24	24	0	3.876324	13346000
KRT8	0	24	24	0	3.876324	11943000
ENO1	0	23	23	0	3.819188	17541000
PLEC	0	23	23	0	3.819188	142280
RPL24	0	21	21	0	3.697645	59896000
RPS3A	0	21	21	0	3.697645	15203000
RPS2	0	21	21	0	3.697645	12651000
DYNC1H1	0	21	21	0	3.697645	331010
RPL26	0	20	20	0	3.632805	70146000
KRT18	0	20	20	0	3.632805	6822400
